# The impact of elevated sulfur and nitrogen levels on cadmium tolerance in *Euglena* species

**DOI:** 10.1038/s41598-024-61964-w

**Published:** 2024-05-22

**Authors:** Victoria Kennedy, Emma Kaszecki, Michael E. Donaldson, Barry J. Saville

**Affiliations:** 1 Environmental and Life Sciences Graduate Program, Trent University, Peterborough, ON Canada; 2 Forensic Science Department, Trent University, Peterborough, ON Canada

**Keywords:** Microbiology, Molecular biology, Environmental sciences

## Abstract

Heavy metal (HM) pollution threatens human and ecosystem health. Current methods for remediating water contaminated with HMs are expensive and have limited effect. Therefore, bioremediation is being investigated as an environmentally and economically viable alternative. Freshwater protists *Euglena gracilis* and *Euglena mutabilis* were investigated for their tolerance to cadmium (Cd). A greater increase in cell numbers under Cd stress was noted for *E. mutabilis* but only *E. gracilis* showed an increase in Cd tolerance following pre-treatment with elevated concentrations of S or N. To gain insight regarding the nature of the increased tolerance RNA-sequencing was carried out on *E. gracilis*. This revealed transcript level changes among pretreated cells, and additional differences among cells exposed to CdCl_2_. Gene ontology (GO) enrichment analysis reflected changes in S and N metabolism, transmembrane transport, stress response, and physiological processes related to metal binding. Identifying these changes enhances our understanding of how these organisms adapt to HM polluted environments and allows us to target development of future pre-treatments to enhance the use of *E. gracilis* in bioremediation relating to heavy metals.

## Introduction

Mining runoff and tailing ponds directly pollute nearby water sources and soil, while aerosolized particles extend the perimeter of pollution to further waterbodies and soil^1^. This contamination has devastating impacts on both ecosystem and human health^1,2^. Cd is one of the most toxic heavy metals (HMs)^3^, with prolonged exposure resulting in decreased chlorophyll content in microalgae^4–6^ and reduced photosynthesis and mitochondrial function in plants^7,8^.

Current methods to remediate areas that are polluted by HMs include chemical precipitation, filtration systems, resin-based ion exchange, flotations, coagulation, and the use of activated carbons^9^. Although these methods are the current industry standard, they are expensive, require a substantial amount of energy and are largely ineffective^10^. On average, these methods remove less than 100 mg/L of HMs from water, and there is considerable variation depending on the landscape of the waterbody^10,11^. Due to these drawbacks, research has focused on removing HMs using bioremediation. Bioremediation is the process of using living organisms to remove contaminants and pollutants from the environment^12^. While there are various plant and fungal species that have shown a natural tolerance and ability to absorb Cd, *Euglena gracilis* has emerged as a novel and more effective tool for the bioremediation of HMs including Cd^13^.

*E. gracilis* is a single-celled, freshwater protist that can grow under photoautotrophic, heterotrophic and mixotrophic conditions^13,14^. Unlike most organisms, *E. gracilis* can survive in a variety of harsh environments, notably those that are acidic (pH 2.5–7) and contain high concentrations of HMs. One example of this type of environment is mine runoffs, where *E. gracilis* can be naturally found^14,15^. *E. gracilis* is capable of absorbing HMs including Cu, Pb, Hg, and Cd, and has demonstrated greater absorption capabilities than other bioremediation organisms^13,15,16^. For example, *Ballota hirsuta*, a common plant in mining regions, demonstrated an absorption capacity of approximately 1.7 × 10^−2^ mg/g for Ni and 1.2 × 10^−2^ mg/g for Cu. In contrast, *E. gracilis* has been shown to absorb 50.1 mg/g of Ni and 1.55 × 10^−2^ mg/g of Cu under similar conditions^17,18^. When grown anaerobically, *E. gracilis* cells displayed higher Cd biosorption capabilities and have been shown to become Cd resistant after several generations of growth exposed to Cd^19^. Recent metabolomic analysis identified metal-binding compounds produced by *E. gracilis* that are enriched in S and N^20,21^. More specifically, five out of six Hg binding compounds identified were found to contain N and classified as polyphenols and lignin monomers, which exhibit metal-binding properties ^20,21^. Interestingly, these compounds were also identified in the related *E. mutabilis*^21^.

*E. mutabilis* is an extremophilic Euglenoid often found in highly toxic environments including acid mine drainage^22^. It is often associated with other microorganisms to form biofilms, and it is believed that these microbial interactions strengthen *E. mutabilis*’ ability to tolerate extreme environments^23^. An *E. mutabilis* culture obtained with a naturally associated yeast from northern Canada had HM tolerance 10–100 times higher than previously reported algae^23^. More recently, the Cd tolerance of an *E. mutabilis* fungal-algal–bacterial (FAB) consortium (CPCC 657) was investigated to identify the significance of the microbial partnerships^24^. While investigating the role of the associated organisms in culture, an *Acidiphilium acidophilum* bacteria and a *Talaromyces* sp. fungus, it was discovered that suppressing their growth through antibiotic and antimycotic agents resulted in decreased Cd tolerance of *E. mutabilis* compared to when the culture was left intact^24^. The high tolerance of both these *Euglena sp.* to heavy metals added to the interest in investigating this organism for potential in bioremediation.

Pre-growth (treatment) of plants has also resulted in increased Cd tolerance. Notably, S treatment has been used to enhance Cd tolerance in *Brassica chinensis*^25^. The rationale for these studies was that S assimilation is linked to an increase in thiol-containing compounds such as phytochelatins and glutathione (GSH) which contribute to Cd tolerance, and stabilizes the ascorbate (AsA)-GSH cycle which helped maintain the balance of reactive oxygen species (ROS)^21^. N containing compounds have also been linked to increased Cd tolerance in plants^26^. Doubling the N supply to wheat (20 mM NO_3_^−^) reduced oxidative stress and growth inhibition caused by Cd^26^. The increased N was also able to mitigate the reduction of both chlorophyll and carotenoids, allowing photosynthesis to continue^26^. Under anaerobic conditions, the combination of decreased oxygen with increased cysteine and sulfide levels (Greenblatt and Schiff medium containing 1.5 mM of sulfate) led to lower Cd toxicity with an IC_50_ = 104 + /− 35 μM CdCl_2_^20^. Similar to the anaerobic growth experiments, exposing aerobic cultures of *E. gracilis* to low concentrations of HgCl_2_ for at least 60 generations resulted in permanent metabolic adaptations that enhanced its ability to accumulate CdCl_2_^27^. Unlike *E. gracilis,* the impact of a pretreatment on the Cd tolerance of the FAB consortium within *E. mutabilis* had not been investigated in past work^24^.

The innate HM tolerance and metal-binding properties of *E. gracilis* and *E. mutabilis* allow them to survive in toxic environments such as acid mine drainage and are essential for their potential use as bioremediation tools. Additionally, previous work successfully enhanced the HM tolerance of *Euglena* by manipulating its growth conditions. This information, combined with the discovery of S and N rich polyphenols and lignin monomers, lead to the hypothesis that pre-treatment with elevated levels of S and N could increase the tolerance of *E. gracilis and E. mutabilis* to Cd.

While there have been many studies of *E. gracilis* and HM tolerance, limited research suggests that the HM tolerance of *E. mutabilis* is greater than *E. gracilis*^15^. In this study we assessed the tolerance of *E. gracilis* (CPCC 95), a commercial axenic culture, and *E. mutabilis* (CPCC 657), an environmental FAB consortium, to CdCl_2_ following pre-treatments with elevated S and N. We assessed growth and cell viability of both *Euglena* species to determine the impact of Cd. The data indicate *E. mutabilis* cultures are more tolerant of Cd and that the presence of other organism in the consortium may influence the impact of elevated S and N. In contrast, *E. gracilis* Cd tolerance is increased by S and N pretreatment, and this allowed us to identify functional genes associated with altered transcript levels in response to pre-treatments and Cd exposure using RNA sequencing. The findings of this research improve our understanding of Cd tolerance in *E. gracilis* and *E. mutabilis*, they also provide insight regarding the mechanisms behind HM tolerance in *E. gracilis*. The knowledge gained could enhance the ability to use *Euglena sp.* as tools for bioremediation.

## Results

### Growth of *Euglena gracilis* and *Euglena mutabilis* in pre-treatment media

The impact of elevated S and N on Cd tolerance was assessed by growing cultures of *E. gracilis* and *E. mutabilis* in Modified Acid Medium (MAM) at a pH of 4.3^28,29^, with increased concentrations of MgSO_4_·7H_2_O (13.85 g/L) or the addition of NH_4_NO_3_ (5.85 g/L). Growth assays and microscopy were used to assess the impact of elevated S and N on cell morphology. The 10 times level was selected because this was the greatest concentration that could be used without having a substantial impact on cell morphology especially in the case of N (Fig. S1).

**Figure 1:**
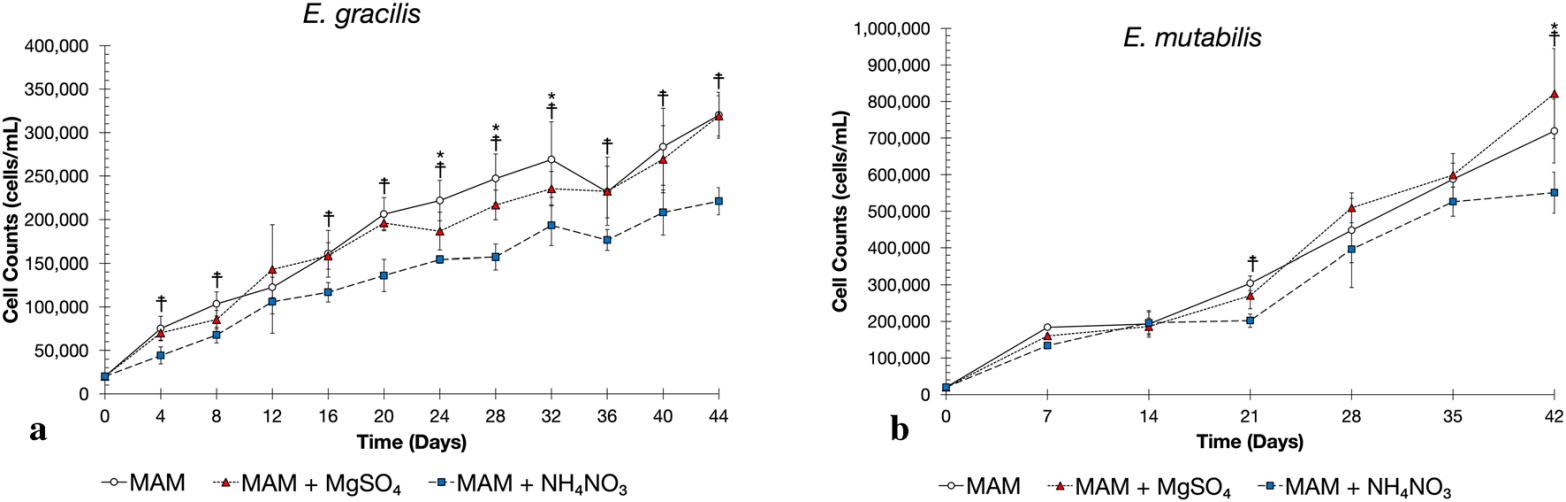
Growth of *Euglena* pretreatment. Growth of *E. gracilis* (**a**) and *E. mutabilis* (**b**) is unaffected by elevated S but decreased with elevated N. 20,000 cells of *E. gracilis* and *E. mutabilis* were inoculated into their treatments and grown for 44 and 42 days, respectively, with *E. gracilis* cells counted and transferred into fresh media every 4 days and *E. mutabilis* cells counted and transferred every 7 days. A one-way ANOVA was used to calculate statistical difference in cell counts of the S (MAM + MgSO_4_·7H_2_O) or N (MAM + NH_3_NO_3_) pretreatment compared to control growth (MAM). The results of the one-way ANOVA for *E. gracilis* cultures are as follows: F-value = 17.37, *p*-value = 7.7 × 10^−9^. The results of a one-way ANOVA for *E. mutabilis* cultures are as follows: F-value = 46.64, *p*-value = 1.8 × 10^−8^. Differences between control cells and cells in the S pretreatment are denoted by an asterixis (*; *p* =  < 0.05), while differences between control cells and cells in the N treatment are denoted by a dagger (☨; *p* =  < 0.05). Error bars represent standard deviation in viable cell counts between biological replicates (*n* = 7).

Cell counts of *E. gracilis* grown in MAM (Control) and MAM with elevated S (S-treatment) and N (N-treatment) were reported every 4 days for 44 days, while *E. mutabilis* were reported every 7 days for 42 days (Fig. 1). The difference in sampling intervals were selected to be consistent with previous research using these organisms which suggested that *E. mutabilis* grows slower than *E. gracilis*^15,27,30^, however our findings contradict these reports. A linear regression performed on the growth curves of Control, S-treated and N-treated *E. gracilis*, generated growth rates (slope) of 6.2 × 10^3^ cells/mL per day (R^2^ = 0.9411; F = 1.79 × 10^−7^), 5.9 × 10^3^ cells/mL per day (R^2^ = 0.9546; F = 4.82 × 10^−8^), and 4.4 × 10^3^ cells/mL per day (R^2^ = 0.9617; F = 2.05 × 10^−8^), respectively. In contrast, the growth rates of Control, S-treated and N-treated *E. mutabili*s, are 2.6-fold (16.1 × 10^3^ cells/mL per day; R^2^ = 0.9755; F = 3.22 × 10^−5^), 3.1-fold (18.4 × 10^3^ cells/mL per day; R^2^ = 0.9555; F = 1.44 × 10^−4^), and 3.0-fold (13.12 × 10^3^ cells/mL per day; R^2^ = 0.9530; F = 1.65 × 10^−4^) higher, respectively. A one-way ANOVA comparing growth between pretreatments also identified significant differences in cell counts. These differences are observed in *E. gracilis* and *E. mubtabilis* when comparing cell counts in S-treated and N-treated cultures to the cell counts in the Control cultures.

### ***Euglena gracilis*** and ***Euglena mutabilis*** cell viability after exposure to 25 $\mu M$ CdCl_2_

**Figure 2:**
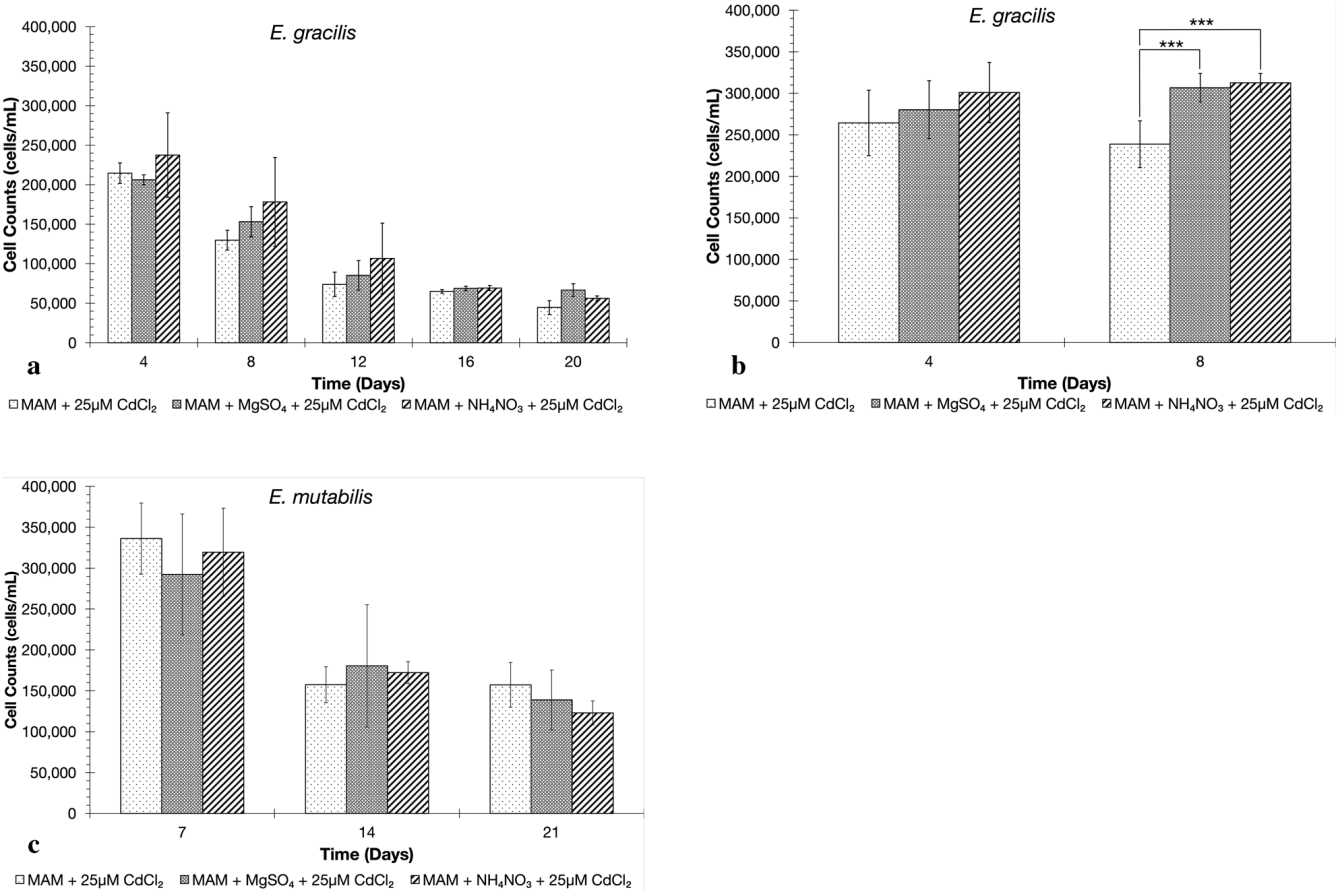
Assessment of Cd tolerance of *Euglena gracilis and Euglena mutabiilis*. *E gracilis* exhibits increased Cd tolerance following S-treatment and N-treatment during two separate time course exposure to 25 μM CdCl_2_ with separate biological replicates(**a**,**b**) In contrast, the Cd tolerance of *E. mutabilis* did not appear to increase following S-treatment and N-treatment (**c**). An 8-day time course for *E. gracilis* (**b**) revealed the viable cell counts remained high enough to provide sufficient biomass for RNA isolation. Bars on the graph represent the number of viable cells in each culture (cells/mL). Asterixis indicate significant difference in cell counts (* = *p* < 0.05, ** = *p* < 0.01, *** = *p* < 0.001) as determined by a one-way ANOVA test across seven biological replicates for *E. gracilis* (Fig. 2b: Day 4 F-value = 1.73; Day 4 *p*-value = 0.2057; Day 8 F-value = 29.13; Day 8 *p*-value = 2.27 × 10^−5^) and three biological replicates for *E. mutabilis*. Error bars represent the standard deviation between viable cell counts of the biological replicates.

Following the pretreatment of Control, S-treatment and N-treatment *E. gracilis* and *E. mutabilis* cultures, the tolerance of *E. gracilis* and *E. mutabilis* cultures to Cd were assessed. Pretreated *E. gracilis* cells were exposed to either control (MAM only) or Cd (MAM + 25 μM CdCl_2_) conditions with cell counts every 4 days for 20 days, while pretreated *E. mutabilis* cells were exposed to control and Cd conditions with cell counts every 7 days for 21 days (Fig. 2). The concentration of CdCl_2_ was selected following preliminary tests which showed that 25 μM CdCl_2_ elicited inhibitory growth responses from *E. gracilis* without causing extensive cell death (Fig. S2). Preliminary testing also indicated that an 8-day period of exposure to Cd not only elicited a detectable response as observed in the number of viable cells present but provided an adequate number of cells for RNA isolations and RNA-sequencing across all treatment conditions (Fig. 2a; Fig. S2).

Pre-treating *E. gracilis* cultures with elevated concentrations of S and N prior to CdCl_2_ exposure resulted in higher cell counts, after 8 days, relative to non-pretreated cultures (Fig. 2a,b). In contrast, pretreated *E. mutabilis* cultures did not show a consistent or statistically significant difference in viable cells relative to non-pretreated cells after 21 days of CdCl_2_ exposure (Fig. 2b). The lack of differences in response seen for *E. mutabilis* could be a result of interactions between members of the FAB consortium or due to metabolism of the bacteria, fungus, or both. Neither the potential interactions, nor the effects of individual organisms in the consortium were assessed. Although *E. mutabilis* cultures exhibited a varied response following CdCl_2_ exposure, they also showed higher cell counts than the *E. gracilis* cultures after 14 and 21 days. Control *E. mutabilis* cultures contained 3.5 times the cells in the presence of CdCl_2_, 1.9 times with the S-treatment cultures, and 1.8 times the number of viable cells with the N-treatment cultures, relative to the 20-day *E. gracilis* cultures (Fig. 2a,c). This indicated that only *E. gracilis* responded to pre-treatment but that *E. mutabilis* was the more Cd tolerant culture.

The impact of pre-treatment on *E. gracilis* prompted an investigation, using RNA-sequencing, to determine which genes were responding to cadmium exposure, and if there are differences in gene transcript levels in the S-treatment and N-treatment cultures compared to the Control cultures. The time course was repeated for a shortened 8 days and at this point there was a significant difference in cell counts between the pretreated and non-pretreated cultures and there was enough biomass for reliable RNA isolation (Fig. 2b). Cultures from an 8-day time course were used for RNA isolation.

Trypan Blue Solution (Sigma-Aldrich, Mississauga) was used for cell viability assessments in the *E. gracilis* and *E. mutabilis* cultures (Table 1). While there were fewer *E. gracilis* cells present in the cultures exposed to CdCl_2_, the fraction of dead cells was higher in the *E. mutabilis* cultures. Assessing the differences in cell growth and cell viability between species indicated that only *E. gracilis* had increased CdCl_2_ tolerance following pre-treatment with S or N. This data further supported the selection of *E. gracilis* to investigate the impact of S and N pre-treatment on gene expression change and the selection of the 8-day time point to ensure a statistical difference in growth of pre-treated cultures and sufficient biomass for RNA isolation.

**Table 1:** Comparison between the average number of *E. gracilis* and *E. mutabilis* cells in each treatment on the final day of 25 μM CdCl_2_ exposure, 20 and 21 days, respectively, and the frequency of dead cells in each treatment.

	*Euglena gracilis* (day 20)		*Euglena mutabilis* (day 21)		*Euglena gracilis* (day 8)	
Treatment	Total cell count (cells/mL)	Frequency of dead cells (%)	Total cell count (cells/mL)	Frequency of dead cells (%)	Total cell count (cells/mL)	Frequency of dead cells (%)
Control	44,646	2.27	184,957	17.59	241,963	1.35
S-Pretreated	66,666	0	183,221	31.92	306,785	0
N-Pretreated	56,250	0	172,749	40.54	313,451	0.28

### RNA-seq, differential gene expression, and gene ontology term analysis

RNA-sequencing was carried out on *E. gracilis* cultures grown in six conditions: Control cultures, S-treatment cultures, and N-treatment cultures each with and without exposure to 25 μM CdCl_2_. For each growth condition six biological replicates were sequenced for a total of 36 samples with an average of 23.3 million raw paired end (PE) reads per library (Table S1). Following the removal of adapter and poor-quality sequences there was an average of 19.5 million trimmed PE reads per library. A de novo transcriptome was assembled using a combined total of 117.3 million trimmed PE reads. Once completed, the transfrag assembly represented 838,537 transcripts with an N50 value of 892 and these transcripts were from 459,533 *Euglena gracilis* genes. BUSCO analysis comparing *E. gracilis* transfrag assembly to the *eukaryota* Orthodb v10 orthologs showed 82.8% of orthologs within the database were found in the *E. gracilis *de novo assembly. An average of 23.2 million trimmed paired-end reads were aligned to the de novo transcriptome assembly across all libraries (Table S2).

**Figure 3:**
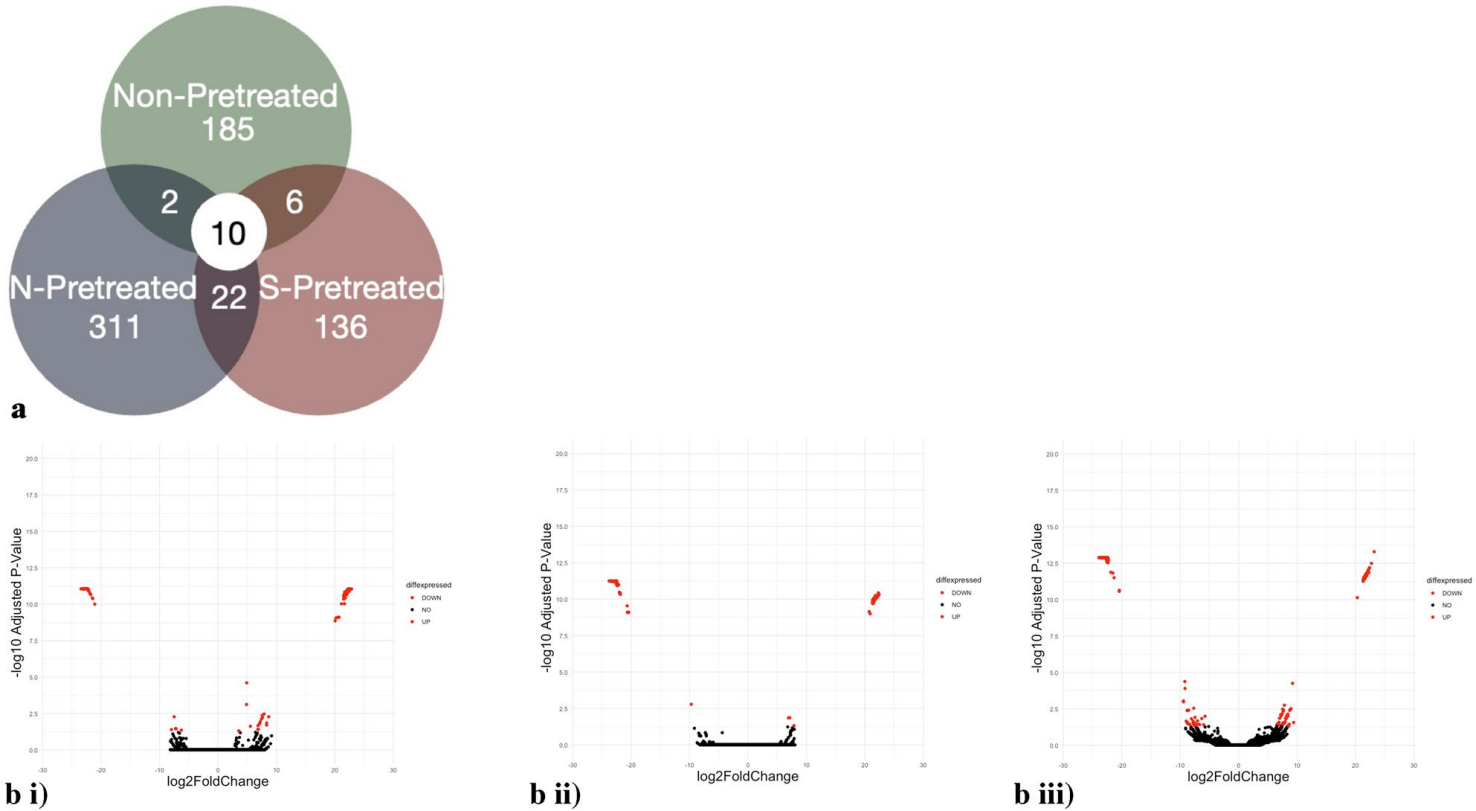
Distinct gene sets have altered transcript levels in cultures with different pretreatments. (**a**) Venn diagram of Differentially expressed genes between non-pretreated *Euglena gracilis* cultures (green), N pretreated *Euglena gracilis* cultures (blue) and S pretreated *Euglena gracilis* cultures (red) in the presence of CdCl_2_. (**b**) volcano plots comparing the -log10 adjusted *p*-value (y-axis) to the log2 fold change (x- axis) between non-pretreated cultures with and without CdCl_2_, (**c**) Volcano plots comparing the -log10 adjusted *p*-value (y-axis) to the log2 fold change (x- axis) between S pretreated cultures vs non-pretreated cultures with CdCl_2_and d) Volcano plots comparing the -log10 adjusted *p*-value (y-axis) to the log2 fold change (x- axis) between N pretreated cultures vs non-pretreated cultures with CdCl_2_. Red dots indicate genes that are differentially expressed (log2 fold change of > 2 or < -2) and are statistically significant (*p* < 0.05), while black dots indicate genes whose expression was not significantly different.

Differential gene expression (DGE) analysis of control (MAM only), S-pretreated, and N-pretreated *E. gracilis* cultures exposed to 25 μM CdCl_2_ identified 185, 136, and 311 unique transcripts, respectively (Fig. 3a)^31^. DGE analysis identified only 10 transcripts that were shared among the control and pretreatment cultures, and 22 shared transcripts between S and N pretreated cultures. Comparisons between Control cultures that have not been exposed to CdCl_2_ and those that have revealed a greater number of up regulated transcripts than down regulated transcripts (Fig. 3b). In contrast, when comparing Control cultures exposed to CdCl_2_ with S-treatment cultures exposed to CdCl_2_ (Fig. 3c) there were more down regulated transcripts. While comparisons between Control cultures exposed to CdCl_2_ and N-treatment cultures exposed to CdCl_2_ exhibit similar numbers of up and down regulated transcripts (Fig. 3d).

To identify the potential genes encoding proteins that were associated with these transcript level changes, a blastx search was conducted using the NCBI non-redundant protein and SWISS-Prot databases, as well as the Ensembl databases for *Arabidopsis thaliana*, *Chlamydomonas reinhardtii*, *Synechocystis sp., Homo sapiens*, and *Trypanosoma brucei*. There is no complete and annotated genome for *E. gracilis*, therefore to identify potential functions of proteins encoded by transcripts we used well-annotated databases. The blastx results of the DGE analysis identified four main classes of genes encoding proteins that showed transcript level changes: transmembrane transport, stress response, metabolism, and metal binding^31^.

S-treatment and N-treatment cultures revealed differences in transcript expression related to respiration, including NADH dehydrogenase and NADH-ubiquinone oxidoreductase. These transcripts exhibited decreased expression in S-treatment cultures without CdCl_2_ exposure compared to control cultures that were not exposed to CdCl_2_, and in N-treatment cultures that were not exposed to CdCl_2_ compared to N-treatment cultures that were. Conversely, transcript expression related to respiration were increased in all N-treatment cultures, either subsequently exposed to CdCl_2_ or not, compared to Control cultures without CdCl_2_ exposure. S-treatment and N-treatment cultures exposed to CdCl_2_ revealed decreased ABC-transporter protein expression compared to Control cultures exposed to CdCl_2_. Additionally, Control cultures exposed CdCl_2_ showed elevated levels of transcripts for genes encoding chemotaxis proteins. The chemotaxis protein transcripts showed decreased expression in S-treatment and N-treatment cultures exposed CdCl_2_, while transcripts encoding genes for heat shock proteins were increased in these treatments. These findings illustrate transcript level differences as a result of the S-treatment and N-treatment of *E. gracilis* prior to CdCl_2_ exposure. Proteins related to metabolism also vary based on pretreatment. S-treated cultures exposed to CdCl_2_ exhibited a decrease in transcript levels for genes encoding cystathionine gamma lyase and cystathionine beta synthase, while N-treated cultures exposed to CdCl_2_ exhibited a decrease in transcript levels for genes encoding threonine dehydratase and an increase in transcript levels for genes encoding 2-isopropylmalate synthase. N-treated cultures also revealed transcript level changes for genes encoding proteins related to metal binding. This included an increase in calmodulin and serine/threonine kinase, and a decrease in phospholipid transporting ATPase and L-ascorbate peroxidase.

Further analysis of gene ontology (GO) terms using the PANTHER classification system revealed enrichment of GO terms which were consistent with DGE analysis results including cysteine metabolic process, ABC-type transporter activity, iron-S cluster binding, and metal ion binding (Table 2). Several GO-terms of interest were identified using data from the model microalgae *Chlamydomonas reinhardtii*, parasitic kinetoplastid *Trypanosoma brucei*, and the model organism *Arabidopsis thaliana* (Table S3). Moreover, significantly enriched GO terms emerge when evaluating cultures that have been exposed to Cd which may signify their role in Cd tolerance.

**Table 2:** Select results for GO term results on non-pretreated, S pretreated and N pretreated Euglena gracilis cultures after exposure to 25 μM CdCl_2_.

		Annotation Set	Fold-Enrichment	*p*-value	Database
GO-Term non-pretreated exposed to CdCl_2_ versus S-pretreated exposed to CdCl_2_	Cysteine metabolic process (GO:0,006,534)	Biological process	> 100	9.79 E−6	*Trypanosoma brucei*
Non-pretreated exposed to CdCl_2_ versus N-pretreated exposed to CdCl_2_	ABC-type transporter activity (GO:0,140,359)	Molecular function	40.65	6.28E −5	*Chlamydomonas reinhardtii*
N-pretreated exposed to CdCl_2_ versus N-pretreated not exposed to CdCl_2_	Iron-sulfur cluster binding (GO:0,051,536)	Molecular function	23.88	2.46E−4	*Chlamydomonas reinhardtii*
	Metal ion binding (GO:0,046,872)	Molecular function	4.25	3.53E−4	*Chlamydomonas reinhardtii*

### RT-qPCR

**Figure 4:**
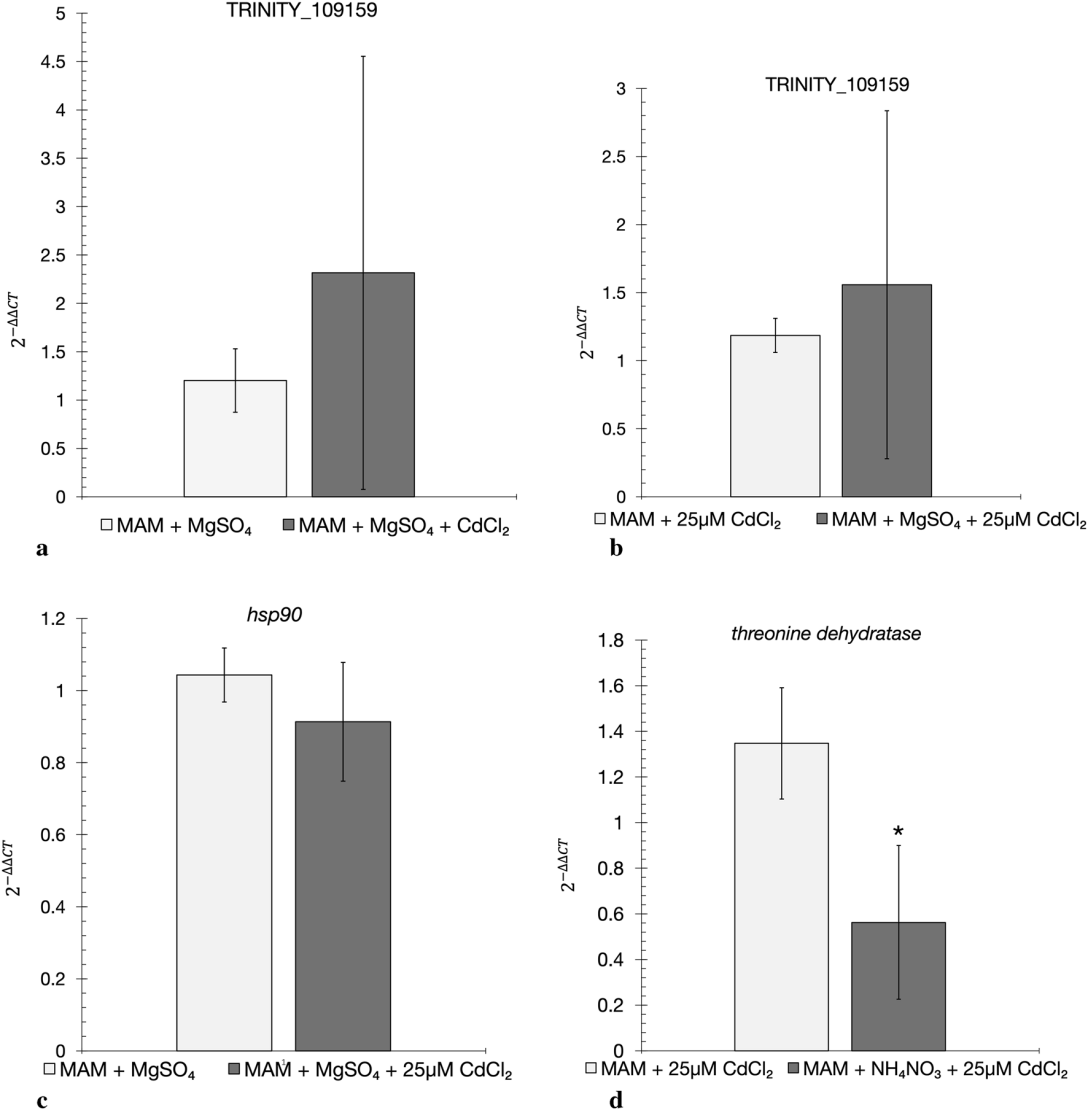
RT-qPCR supports trends in expression noted in RNA-sequencing. Transcript levels, determined by RT-qPCR of: (**a**) TRINITY_109159 compared in non-pretreated and S pretreated *E. gracilis* cultures exposed to CdCl_2_, (**b**) transcript TRINITY_109159 compared in S pretreated cultures in the presence and absence of CdCl_2_, (**c**) transcript encoding a potential *hsp90* protein compared in S pretreated cultures in the presence and absence of CdCl_2_, and (**d**) transcript encoding threonine dehydratase compared in N pretreated *E. gracilis* cultures in the presence and absence of CdCl_2_. RT-qPCR was performed using 3 technical replicates using the housekeeping genes *ef1* and *actin* for normalization. The ${2}^{-\Delta \Delta Ct}$ method was used to determine relative expression using either the pretreated culture without CdCl_2_, or the non-pretreated culture exposed to 25 μM CdCl_2_ as the reference**.** Error bars represent the standard deviation between the biological replicates (*n* = 4 except for *hsp90* where *n* = 3), while a star (*) denotes statistical significance using a t-test (F-value = 0.52, *p*-value = 0.017).

RT-qPCR was carried out on RNA isolated from *E. gracilis* cells under the following treatment conditions: Control without CdCl_2_ exposure, Control with 25 μM CdCl_2_ exposure, S-treated without CdCl_2_ exposure, S-treated with 25 μM CdCl_2_ exposure, N-treated without CdCl_2_ exposure, and N-treated with 25 μM CdCl_2_ exposure. Transcripts were selected for RT-qPCR based on relevant biological functions of predicted proteins, or degree of transcript level change as indicated by DGE. The RT-qPCR results indicate that the transcripts TRINITY_109159, *hsp90*, and threonine dehydratase showed similar trends in expression level changes when compared to the results obtained with RNA-sequencing (Fig. 4). RNA-sequencing results for TRINITY_109159 indicate an increase in S-treated cultures that were exposed to CdCl_2_, but a decrease in S-treated cultures not exposed to CdCl_2_. This is an identification of a novel gene transcript since it has no sequence similarity in any database. RT-qPCR results also supported the detection of increased transcript levels in S-treated cultures that were exposed to CdCl_2_ relative to control (MAM only) cultures, and S-treated cultures that were not exposed to CdCl_2_. Notably, RNA-sequencing indicated an increase in the transcript for the *hsp90* gene in S-treated cultures that were exposed to CdCl_2_ when compared to S-treated cultures that were not exposed to CdCl_2_. The RT-qPCR results further showed an increase in the transcript level for the *hsp90* gene in S-treated cultures exposed to CdCl_2_, although DGE analysis did not indicate the same level of increase. Finally, DGE analysis identified a decrease in the level of transcripts for a gene encoding threonine dehydratase in N-treated cultures that were exposed to CdCl_2,_ a decrease was also noted for this gene in the RT-qPCR. The RT-qPCR analyses generally supported the RNA-sequencing results and uncovered differential expression of genes that did not meet the minimum threshold requirements of RNA sequencing DGE analysis.

## Discussion

The tolerance of *Euglena* species to CdCl_2_ and the impact of pre-treatment in elevated S and N levels were investigated. The initial experiments assessed the overall impact of a pretreatment with 5, 10 and 20 times the amount of S (MgSO_4_·7H_2_O) or N (NH_4_NO_3_) compared to control media on the growth and impact on cell morphology for *E. gracilis*. From these results we selected the 10 times level since it was the highest concentration that did not have an impact on cell morphology. In the comparison analyses 10 times the amount of S (60 mM MgSO_4_·7H_2_O) or N (76 mM NH_4_NO_3_) were used and the growth was assessed over a period of 44 days for *E. gracilis* and 42 days for *E. mutabilis* (Fig. 1). At several time points in the assay, growth of S-treatment *E. gracilis* cultures was significantly greater than in control conditions. These findings are consistent with previous work indicating that *E. gracilis* is capable of taking up sulfate from its environment ^32^. Conversely, growth of *E. gracilis* in the N-treatment cultures was significantly decreased over 44 days compared to the control. Although ammonium is the optimal source of N for *E. gracilis*^33,34^ it is unable to use nitrate, nitrite, or urea as a source of N despite still being able to grow in the presence of nitrate^33–35^. Growth in the presence of nitrate at high concentrations could result in reduced growth. There was little or no influence of the S-treatment or N-treatment on the growth of the *E. mutabilis* consortium.

Following the S-treatment and N-treatment, *E. gracilis* and *E. mutabilis* cultures were exposed to 25 μM CdCl_2_ and the impact on growth was assessed (Fig. 2). The significant increases in the number of viable *E. gracilis* cells exposed to CdCl_2_ in the S-treated and N-treated cultures compared to the Control cultures exposed to CdCl_2_ suggest that these treatments enhance Cd tolerance (Fig. 2b). This result resembles the induced higher tolerance to Cd in *E. gracilis* upon pretreatment with Hg^27^. The enhanced tolerance was also noted through the viability assays in which S-treatment and N-treatment led to fewer dead cells compared to the Control cultures (Table 1), and the minimal number of dead *E. gracilis* cells after 20 days exposed to Cd also reaffirm its innate tolerance^4,19^. In contrast, the number of viable *E. mutabilis* cells upon challenge with CdCl_2_ following either S-treatment or N-treatment, were decreased compared to Control cultures (Fig. 2c). The increased tolerance of *E. mutabilis* cultures to 25 CdCl_2_ μM was revealed by the presence of higher cell counts at days 14 and 21 relative to the comparably grown *E. gracilis* cultures. In addition to exhibiting a greater number of viable cells, *E. mutabilis* also exhibits a greater number of dead cells under each treatment. It has been reported that *E. mutabilis* has a greater tolerance to Cd than *E. gracilis*^15^, however the mechanisms that permit this increased tolerance over *E. gracilis* are unknown. It is uncommon that *E. mutabilis* cultures obtained from nature are axenic and the isolates obtained with co-cultured organisms cannot be made axenic^23^. It is possible that the presence of the associate bacteria and fungi in the *E. mutabilis* co-culture contribute to Cd tolerance and moderate nutritional impacts of *Euglena*. Recent work with this consortium revealed that suppressing the growth of the association organisms significantly reduced *E. mutabilis*’ tolerance to Cd^24^. Despite the mechanisms for Cd tolerance remaining unknown, our study indicates that Cd tolerance of *E. mutabilis* is not enhanced by pre-treatment with S and N, unlike *E. gracilis*. To elucidate the molecular changes in *Euglena* as a result of S-treatment and N-treatment and subsequent Cd exposure, we proceeded with RNA-sequencing analysis on *E. gracilis*.

DGE analysis following RNA-sequencing was used to identify genes whose transcript levels are altered in response to the S-treatment and N-treatment as well as to CdCl_2_ exposure (Fig. 3; Table 2). RT-qPCR was used as an alternative approach to assess DGE. There was variation in RT-qPCR results among the four biological replicates (Fig. 4). Challenges with RT-qPCR using *E. gracilis* have been noted by others^36,37^. A common issue is the difficulty in designing primers due to the lack of an annotated reference genome and sufficient databases. Here the RNA sequencing data was used in primer design however, it is possible that primers designed to be highly specific may still amplify other previously unidentified gene family members, and this would skew the detection of transcript level changes from a given family member. Therefore, the level of a target transcripts identified using RNA-sequencing may not be properly assessed using RT-qPCR. Despite this potential issue, RT-PCR results generally supported the trends of expression seen in the RNA-sequencing data. This work provides a comprehensive analysis of *E. gracilis* genes related to Cd tolerance and provides insight for future gene-specific analyses related to metal tolerance and stress.

Our results indicate that Cd tolerance within *E. gracilis* is a dynamic process with various genes being expressed with each pretreatment and under CdCl_2_ exposure. Blastx searches following DGE analysis were used to identify which protein coding genes had altered transcript levels for each treatment condition^31^, genes were selected for additional analysis based on identified functions associated with heavy metal tolerance. These included genes encoding proteins related to respiration, transmembrane transport, stress response, metabolism, and metal binding. The discussion of genes encoding proteins related to respiration and transmembrane transport are located in the supplementary material (Table S4). The following discussion focuses on the impact of both pretreatments and Cd exposure on stress response, metabolism and metal binding.

Our results identified transcript level changes for genes encoding chemotaxis proteins and heatshock proteins. In metal tolerant bacteria, chemotactic responses are altered by the differential expression of chemotaxis proteins upon exposure to Cd^38,39^. In Control cultures exposed to CdCl_2_, there was an increase in transcript levels for a gene encoding a potential chemotaxis protein. The transcript level from this chemotaxis related gene was decreased in S-treated and N-treated cultures, even though Cd tolerance was increased. This may indicate that the S-treatment and N-treatment enhance other tolerance mechanisms and suppress potential tolerance effects related to chemotaxis.

In plants, small heat shock proteins work to protect photosynthesis and electron transport during a variety of stressors, including heavy metal exposure^40^. In *E. gracilis*, proteomic analyses revealed increased expression of *hsp90*, *hsp70*, *hsp55*, and *hsp40* upon exposure to Cd^41,42^. Consistent with this, our results showed an increase in transcript levels for genes encoding *hsp90* and *hsp70* related proteins in control and S-treated cultures, although *hsp70* was decreased in N-treated cultures that were and were not exposed to Cd. This may suggest that growth in elevated N environments causes stress in *E. gracilis*. Interestingly, previous research showed that pre-exposure of *E. gracilis* to low concentrations of Hg over many generations resulted in increased Cd tolerance and accumulation^27^ suggesting that *E. gracilis* may have a generalized stress response that is activated by previous stress exposure and maintained during Cd exposure. It is possible that priming of a stress response increases Cd tolerance.

Transcript analyses of S-treated and N-treated cultures exposed to CdCl_2_ identified transcript level changes for genes related to cysteine metabolic processes and amide biosynthesis, respectively. These transcript level changes were only found when the S-treated and N-treated cultures were subsequently exposed to CdCl_2_, suggesting that the treatments primed *E. gracilis* for subsequent metabolic responses to stress exposure. Previous metabolomic analyses of *E. gracilis* identified increased levels of the S metabolites sulfate, cysteine, and glutathione upon Hg exposure^20^. It has also been suggested that monothiol compounds, rather than phytochelatins, contribute to *E. gracilis*’ tolerance to Cr^6+^^43^. These data suggest a broad range of S based compounds may act as chelators for metals like Cd thereby contributing to the tolerance of *E. gracilis*. The compounds may also be involved in other tolerance-related processes since glutathione can act to mitigate the oxidative stress induced by Cd^44^. Our results showed that S with subsequent CdCl_2_ exposure led to increased transcript levels of cystathionine beta-synthase and cystathionine gamma-lyase, which could increase cysteine synthesis and subsequently the synthesis of metabolites like glutathione. These results are indicative that S-treatment of *E. gracilis* modified and increased cysteine metabolism and could have downstream impacts on the synthesis of glutathione and related metal binding molecules.

Genes related to amide biosynthesis were enriched in *E. gracilis* following Hg exposure^20^, however these biosynthesis genes were not enriched in N-treated cultures. Instead, DGE analysis indicated the downregulation of threonine dehydratase, a key enzyme in branched chain amino acid synthesis, and the upregulation of 2-isopropylmalate synthase, which is involved in leucine biosynthesis and pyruvate metabolism. Threonine dehydratase was also downregulated in the fungus *Psxillud involutus* in response to CdCl_2_ exposure^45^. This suggests a general response of reducing branched chain amino acid synthesis through this pathway under Cd stress. Increased 2-isopropylmalate synthase may compensate for this reduction in branched chain amino acids by increasing leucine production. This, in turn, might alter the TCA cycle by redirecting some pyruvate metabolism. Others have noted that Cd stress can impact amino acid metabolism to suppress the TCA cycle^46^ and past metabolomic studies showed that various stresses alter *E. gracilis* metabolic processes including the synthesis of amino acids^47^. The data presented here supports a model in which N-treatment enhances a shift from the use of threonine dehydratase as the primary catalyst for branched chain amino acid biosynthesis, to utilizing isopropylmalate-synthase. Determining which metabolic impacts of this directly support Cd tolerance requires future investigation.

GO-term analysis revealed that the N-treatment of *E. gracilis* and subsequent CdCl_2_ exposure also enhanced expression of genes involved in “metal cluster binding” and “metal ion binding”. Through DGE we identified decreased expression of L-ascorbate peroxidase and increased expression of calmodulin, which have both been related to metal stress response by playing a key role in mitigating the oxidative stress response in plants^48,49^. Down regulation in *E. gracilis* suggests that the N-treatment suppressed this oxidative stress response. The metal stress induced reactive oxygen species (ROS) must therefore be handled in a different way in *E. gracilis*. In contrast, the elevation of calmodulin is supported by proteomics analyses of *E. gracilis* cultures exposed to heavy metals^42^, suggesting its involvement in *E. gracilis* enhanced Cd tolerance. Calmodulin has been hypothesized to bind Cd as well as Ca due to their chemical similarities^50^. Inhibiting calmodulin caused a decrease in intracellular Cd and a decrease in glutathione and phytochelatin levels in the algae *Ulva compressa*^51^. The enhanced expression of calmodulin in *E. gracilis* following N-treatment could be contributing to the enhanced resistance to Cd. This could occur if calmodulin binds Cd and decreased its concentration in the cytoplasm or if, by binding Cd, calmodulin’s influence on metabolism is altered. Together these data suggest that increased metal binding in response to Cd stress in *E. gracilis* may reflect a more complex response than just binding and sequestering the Cd.

**Figure 5:**
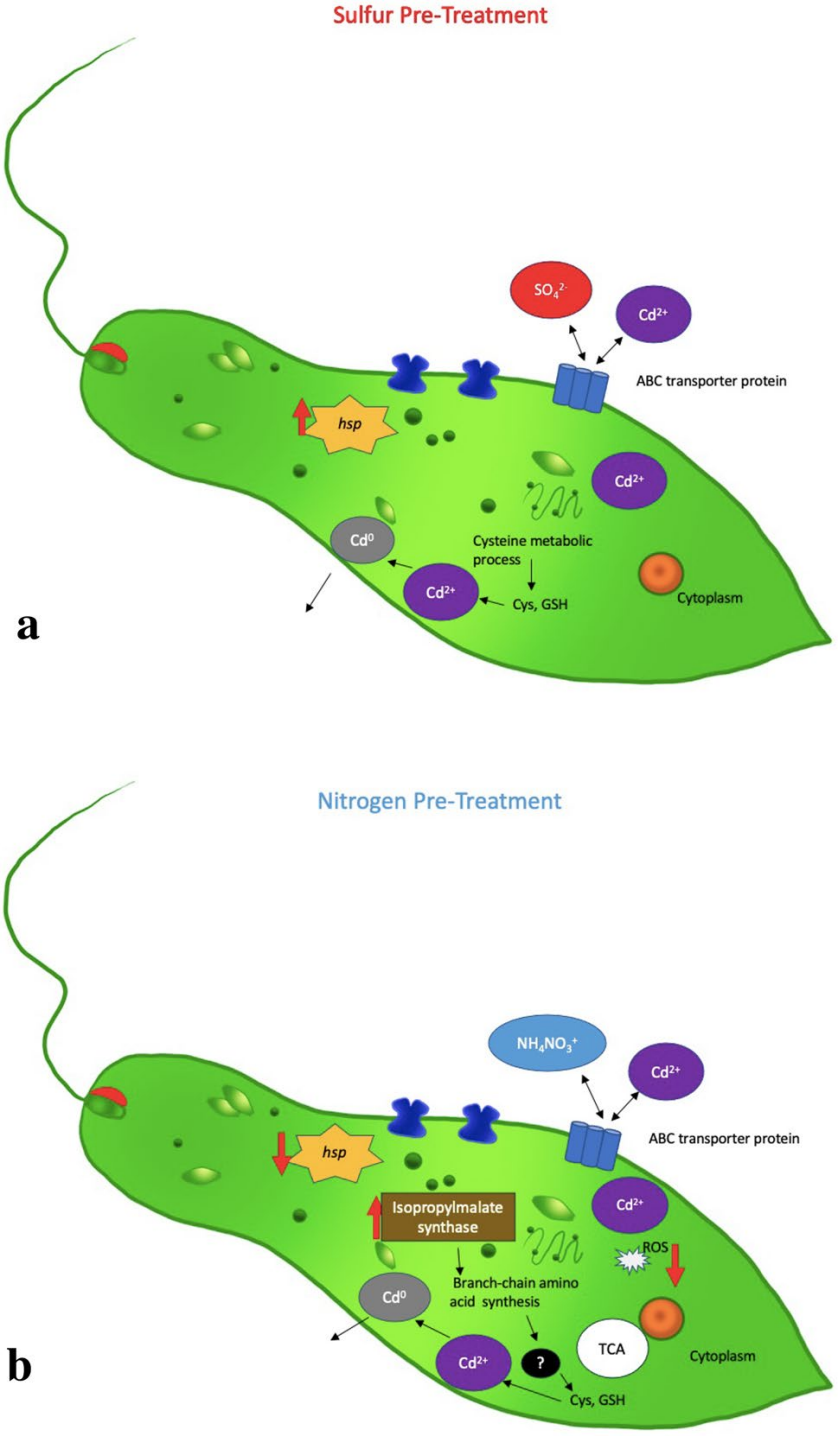
Conceptual model of the transcriptomic responses of S (**a**) and N (**b**) pretreated *Euglena gracilis* cultures after exposure to CdCl_2_. With both pre-treatments, transcript levels were altered for genes involved in transmembrane transport, stress response, metabolic processes, and metal binding. Specifically, genes for distinct ABC and other transporters had altered expression, as did heat shock proteins and potential Cd exporter genes. With the S pre-treatment, GO-terms related to cysteine metabolic processes were enriched after CdCl_2_ exposure. With the N pretreated cultures, the exposure to CdCl_2_ caused a decrease in potential ROS generation and a shift from amine biosynthesis, specifically a decrease in transcript levels for a potential gene encoding threonine dehydratase and an increase in transcript levels for a gene encoding 2-isopropylmalate synthase. Red arrows indicate whether the transcript for the gene-encoding protein was increased or decreased. This model was adapted from Mangel et al. and edited to include the data of the presented research^23^.

The ability to increase Cd tolerance in *E. gracilis* by nutrient shifts is a major finding of the presented work and helps elucidate possible mechanisms by which *E. gracilis* can tolerate Cd (Fig. 5). Data from the comparative RNA-seq analyses enabled us to determine that while the stress of the S-treatment and N-treatment may have triggered a response, the data support the increased resistance being influenced by nutrient specific means. The S-treatment enhanced Cd tolerance by increasing production of glutathione and other compounds that bind Cd and may sequester it. The N-treatment altered branched chain amino acid biosynthesis, leading to increased Cd binding by proteins in *E. gracilis* that do not just sequester the Cd, but may also alter biochemical pathways otherwise involving these binding proteins. The data presented substantially increases the understanding of Cd tolerance by *Euglena sp*. and identifies specific molecular processes involved in *E. gracilis* Cd tolerance. These findings may aid in developing new bioremediation treatment approaches using *E. gracilis.*

## Methods

### Cultivation of *E. gracilis* and *E. mutabilis*

Samples of *E. gracilis* and *E. mutabilis* were collected by the Canadian Phycological Culture Centre (CPCC) located at the University of Waterloo, Ontario, Canada. The strain of *E. gracilis* (CPCC 95) was axenic, while the strain of *E. mutabilis* (CPCC 657) contained fungal and bacterial organisms^24^. Both cultures were grown photoautotrophically shaking at 80 RPM, at a temperature of 22 ± 0.5 °C, and a light regime of 12 h:12 h LD cycle (2691 μMol/m^2^/h) in an environmental chamber (Conviron CMP5090). Stock cultures were grown in Modified Acid Medium (MAM) developed by Olaveson and Stokes (1989): 0.5 g/L of (NH_4_)_2_SO_4_, and MgSO_4_·7H_2_O, 0.3 g/L of KH_2_PO_4_, 0.01 g/L of CaCl_2_·_2_H_2_O, 0.03 g/L of NaCl, 0.01 g/L of Na_2_EDTA·2H_2_O, 0.00498 g/L of FeSO_4_·7H_2_O with 0.00000843 g/L of H_2_SO_4_. The media also included a trace metal solution which when added resulted in final concentrations in MAM of: 2.86 µg/L of H_3_BO_3_, 1.81 µg/L of MnCl_2_·4H_2_O, 0.22 µg/L of ZnSO_4_·7H_2_O, 0.39 µg/L of Na_2_MoO_4_·2H_2_O, 0.079 µg/L of CuSO_4_·5H_2_O and 0.0494 µg/L of Co(NO_3_)_2_·6H_2_O. Since *Euglena* exhibits the highest growth rates in acidic environments^28^ and growth between pH 3.0–5.5 is a common practice that has no negative impacts on *Euglena*’s ability to tolerate HMs, the pH of MAM was adjusted to 4.3^13,29,52–54^ using HCl prior to autoclaving. The pH was not measured while *E. gracilis* and *E. mutabilis* were growing in MAM during experimentation. During that time period no precipitate was observed in the media. After the media was autoclaved, a vitamin stock was added to give a final concentration of 10 µg/L of Vitamin B12, 0.001 µg/L of Biotin and 0.002 g/L of Thiamine HCl in the media. Since MgSO_4_·7H_2_O is an ingredient in MAM its concentration was adjusted to create the S-treatment. The media was modified to include a total of 13.85 g/L of MgSO_4_·7H_2_O to create an environment with 10 times the amount of S (60 mM). Previous research suggests that the optimal N source for *E. gracilis* is ammonium^33,35^. For that reason, 5.85 g/L of NH_4_NO_3_ was added to the media to create an environment with 10 times the amount of N (76 mM) for the N-treatment. Preliminary experimentation indicated that increased S and N exposure did not negatively impact *E. gracilis* growth overtime (Fig. S1).

### Pre-treatment and CdCl_2_ exposure with ***E. gracilis*** and ***E. mutabilis***

To begin the pre-treatment cycle, stock cultures of *E. gracilis* and *E. mutabilis* were grown and used to initiate the S-treated and N-treated cultures and Control cultures each of which were prepared in triplicate. *E. gracilis* cells were inoculated into their pre-treatment medium at a concentration of 20,000 cells/mL with cell counts performed using a hemocytometer (Hausser Scientific) every 96 h to monitor cell growth for a period of 44 days. Culture growth was promoted by collecting the entire volume of culture by centrifugation and resuspending the cells in fresh pre-treatment media prepared using the same recipe as outlined above at a pH of 4.3. This process was performed every 96 h after cell counts were measured. Similarly, 20,000 cells/mL of *E. mutabilis* cells were inoculated into pre-treatment medium and grown for 42 days with cell counts performed using a hemocytometer every 7 days. On the same day that the cell counts were completed, the remaining cells were centrifuged and resuspended into fresh MAM with either the Control treatment, the S-treatment or the N-treatment at a pH of 4.3. The different growth assays for the two species were a result of the observed slower growth of *E. mutabilis* relative to *E. gracilis*^17,28,30^.

Following the completion of the pre-treatment cycle, cells were inoculated into MAM, or MAM with 25 μM CdCl_2_. For *E. gracilis*, seven replicates of each culture type were prepared to be used for RNA-Seq analysis and four more replicates were prepared to be used for future RT-qPCR analysis. The concentration of cells inoculated into each condition was 200,000 cells/mL. For *E. gracilis*, cell counts were performed using a hemocytometer every 96 h for up to 8 days, at each time point cell viability was also assessed using Trypan Blue Solution (Sigma-Aldrich, Mississauga) staining. Once the cell counts were completed, they were used to normalize cultures to the biological replicate with the lowest concentration of cells and cells were resuspended in fresh media of the appropriate condition. For *E. mutabilis*, all culture types were prepared in triplicate and cell counts, and viability staining were carried out in a similar manner, but the time points were every 7 days for up to 21 days. Cells were observed and counted at 10× magnification using a Zeiss AXIO microscope. At each timepoint, cell counts were used to normalize cultures to the biological replicate with the lowest concentration of cells and cells were resuspended in fresh media similar to the methods used for *E. gracilis*. After the final day in CdCl_2_ the total culture volume was centrifuged, supernatant was removed, and cell pellets were frozen with liquid N_2_ and stored at -80℃ ahead of RNA isolations.

### Statistical analysis

Differences in viable *Euglena* cell counts were identified through a one-way ANOVA (⍺ = 0.05), and if differences were identified (*p* < 0.05) a Tukey–Kramer post-hoc analysis was carried out to elucidate the differences amongst the groups. Differences in transcript expression using RT-qPCR were assessed using a Student’s t-test (⍺ = 0.05).

### RNA isolation and DNase1 treatment

Cells from CPCC 95 *E. gra*cilis were removed from − 80 °C and resuspended in 2 mL of TRIzol™ Reagent (Invitrogen™). Samples were transferred to MP Biomedical Lysing Matrix C tubes and lysed using a MP Biomedical Fast Prep-24. The RNA was precipitated using 250 $\mu$L of 0.8 M disodium citrate/1.2 M NaCl and 250 µL isopropanol. DNA was removed from the samples using a Dnase1 treatment before the RNA was precipitated again using the same RNA precipitation solution that was mentioned above and isopropanol. The quality of each sample was assessed by visualizing RNA following electrophoretic separation on a 1.5% BPTE agarose gel following glyoxal denaturation. Dnase-treated RNA and a ssRNA ladder were loaded on the gels (New England BioLabs, Whitby, Canada).

### cDNA library preparation and RNA-sequencing

22 $\mu$L aliquots of selected Dnase-treated RNA samples were sent to the Centre for Applied Genomics (TCAG) at the Hospital for Sick Children (Toronto, Canada) where they underwent further quality assessment, cDNA library preparation and RNA-sequencing. The quality of each RNA sample was assessed using a BioAnalyzer (Agilent Technologies). The quality of the biological replicates was determined based on the RNA Integrity Number (RIN) and the DV200% with an emphasis being placed on the DV200%. Selected RNA samples underwent Poly(A) enrichment using oligo dT-beads and the subsequent cDNA libraries were prepared using the NEBNext Ultra Directional RNA Library Prep Kit for Illumina (New England BioLabs). Once prepped, the 36 barcoded libraries were pooled and sequenced on two lanes of the NovaSeq (Illumina Inc) system producing paired-end (PE) reads that were 150 bp long.

### Sequence quality assessment, trimming

Fastq files for the 36 sequenced libraries were downloaded using the TCAG data portal command line tool. Sequence quality was assessed using FastQC (v0.11.9)^55^. The generated html files were reviewed to assess Phred scores across all the generated reads for each file. Once the minimum quality was assessed, sequences were trimmed using Trimmomatic (v0.39) with the following specifications: Illuminaclip:TruSeq3-PE-2.fa:2:30:10 sliding window:4:5 leading:5 tailing:5 minlen:25^56^. The trimmed sequences were used in the formation of a single de novo transcriptome assembly of *E. gracilis* using Trinity (v2.12.0) set to ‘strand-specific mode (RF)’^57^. The completeness of the assembly was assessed using BUSCO (v5.2.2) and the ‘eukaryota_odb9’ dataset^58^. Trimmed sequences were aligned to the assembled transcriptome using Bowtie2 (v2.4.2) with the ‘SS_lib_type RF’ feature^59^. Finally, RSEM (v1.3.3) was used to estimate the transfrag abundance for each library based on the settings used by Li & Dewey^60^.

### Differential gene expression and GO-term analysis

DeSeq2 (v1.30.0) was implemented using the SarTools (v1.7.4.0) program to assess the changes in transcript levels^61,62^. Individual 1 on 1 comparisons were completed to assess genes that were differentially expressed based on the pre-treatment of the cultures, and their exposure to CdCl_2_ (Table S1). The analysis was also used to assess the changes in transcript levels of the pretreated *Euglena gracilis* cultures compared to non-pretreated *Euglena gracilis* cells. The statistical model “treatment” was used for the comparison with a false discovery rate of (FDR) < 0.05. DeSeq2 was run with the settings: cooksCutoff = True, independentFiltering = True, alpha = 0.05 to set the threshold for significance, pAdjustMethod = BH (Benjamini/Hochberg *p*-value adjustment method), typeTrans = rlog, and locfunc = median to estimate size factors. The genes that were identified to have transcript level changes according to DESeq2 were characterized using NCBI blast 2.11.0 + blastx to search the NCBI protein databases as well as SWISS-PROT databases (accessed January 2022) using an E-value of 1E-5 to identify genes encoding proteins with similar sequences. The *Arabidopsis thaliana*^20,63,64^, *Chlamydomonas reinhardtii*^20^, *Synechocystis sp*.^20^, *Homo sapiens*^20,63,64^ *and Trypanosoma brucei*^63,65^ databases on Ensembl 90 were searched using blastx to characterize genes that were further analyzed for gene ontology (GO) enrichment analysis (accessed February 2022). These databases were used since there is not a well annotated genome sequence for *E. gracilis*. Using these databases allowed for a comprehensive search for gene similarities and functional identities using well annotated databases covering a variety of species, some of which share similarities to *Euglena sp.* The genes listed were run in the PANTHER gene ontology database (http://pantherdb.org/index.jsp) on the default settings to test for statistical over-representation of the identified GO terms.

### RT-qPCR

For RT-qPCR, cDNA was synthesized from 10 to 15 $\mu$g of Dnase treated RNA using the TaqMan Gold RT-PCR Kit (Applied Biosystems, Connecticut MA, USA). cDNA samples were stored at − 20 °C until they were used for RT-qPCR. Based on the DGE results, select genes were analyzed using RT-qPCR with the Applied Biosystems Power SYBR Green PCR Master Mix (Applied Biosystems). Primers were designed from the partial sequences of the transcripts using Sigma Oligo Architect to ensure minimal to no dimers, and an optimal amplicon size and annealing temperature. Each reaction contained 4 $\mu$L of dH2O, 2 $\mu$L of each primer at a concentration of 5 uM, 10 $\mu$L of Power SYBR Green PCR Master Mix and 2 $\mu$L of cDNA template. RT-qPCR was carried out in a QuantStudio 3 (Applied Biosystems) with the following conditions: hold stage [50 °C for 2 min then 95 °C for 10 min], PCR stage 40 cycles of [95 °C for 15 s, X°C for 1 min], and melt curve stage [95 °C for 15 s, 60 °C for 1 min, 95 °C for 15 s] with X°C referring to the annealing temperature of the specific primer pair (Table S3). Each RT-qPCR run was performed in triplicate. For RT-qPCR the housekeeping genes that were used were a gene encoding actin and a gene encoding elongation factor 1 (*ef1)*. Both housekeeping genes were used as a control for normalization. This analysis was carried out using four biological replicates except in the case of analysis for the transcription encoding *hsp90*, which used only three biological replicates.

### Supplementary Information

Supplementary Information.

## Data Availability

The datasets generated and/or analysed during the current study are available in the NCBI SRA (sequence read archive) repository, PRJNA1024496 (https://www.ncbi.nlm.nih.gov/bioproject/PRJNA1024496).
