# Supplementary material for: The impact of elevated sulfur and nitrogen levels on cadmium tolerance in Euglena species
Source: Sci Rep. 2024 May 22;14:11734. doi: 10.1038/s41598-024-61964-w (PMC11111685; doi:10.1038/s41598-024-61964-w)
Supplement: Supplementary file 1 — Supplementary Information. [file 41598_2024_61964_MOESM1_ESM.pdf]

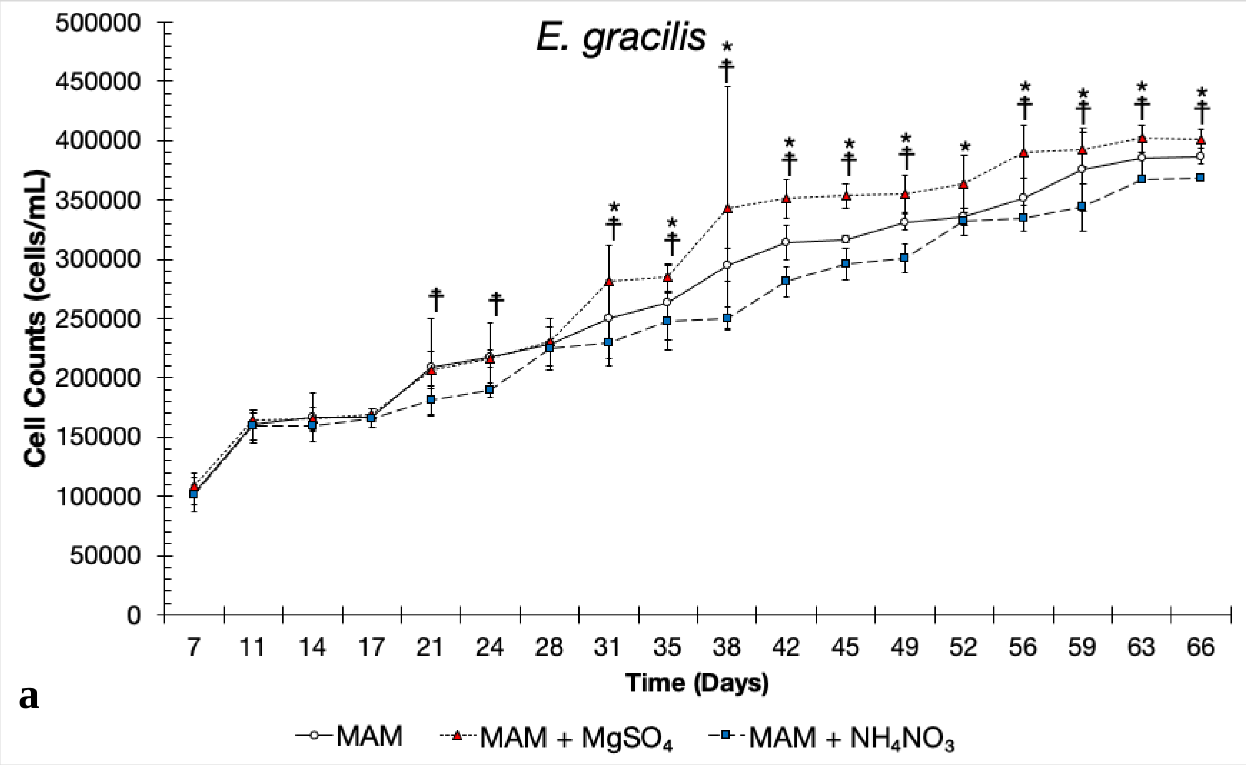

Figure S1: Assessment of growth of *E. gracilis* in pretreatment media. a) Growth of *E. gracilis* in MAM, MAM with elevated S (60 mM; 10x the amount of S compared to control MAM), and MAM with elevated N (76 mM; 10x the amount of N compared to control MAM) over 66 days. A linear regression displayed that while cells grown in elevated N exhibited slower growth rates (slope = 4.3 x103 cells/mL per day; R^2 = 0.9863; F = 2.50 $x10^{-16}$) than control cells (slope = 4.6 x103 cells/mL per day; R^2 = 0.9792; F = 6.81 $x10^{-15}$), cells grown in elevated S exhibited the fastest growth rate (slope = 5.1 x103 cells/mL per day; R^2 = 0.9507; F = 7.03 $x10^{12}$) and ultimately resulted in the greatest increase in cell counts after 66 days. Differences between control cells and cells in the S pretreatment are denoted by an asterixis (*; p = < 0.05), while differences between control cells and cells in the N treatment are denoted by a dagger (†; p = < 0.05). Error bars represent standard deviation in viable cell counts between biological replicates (n = 3).

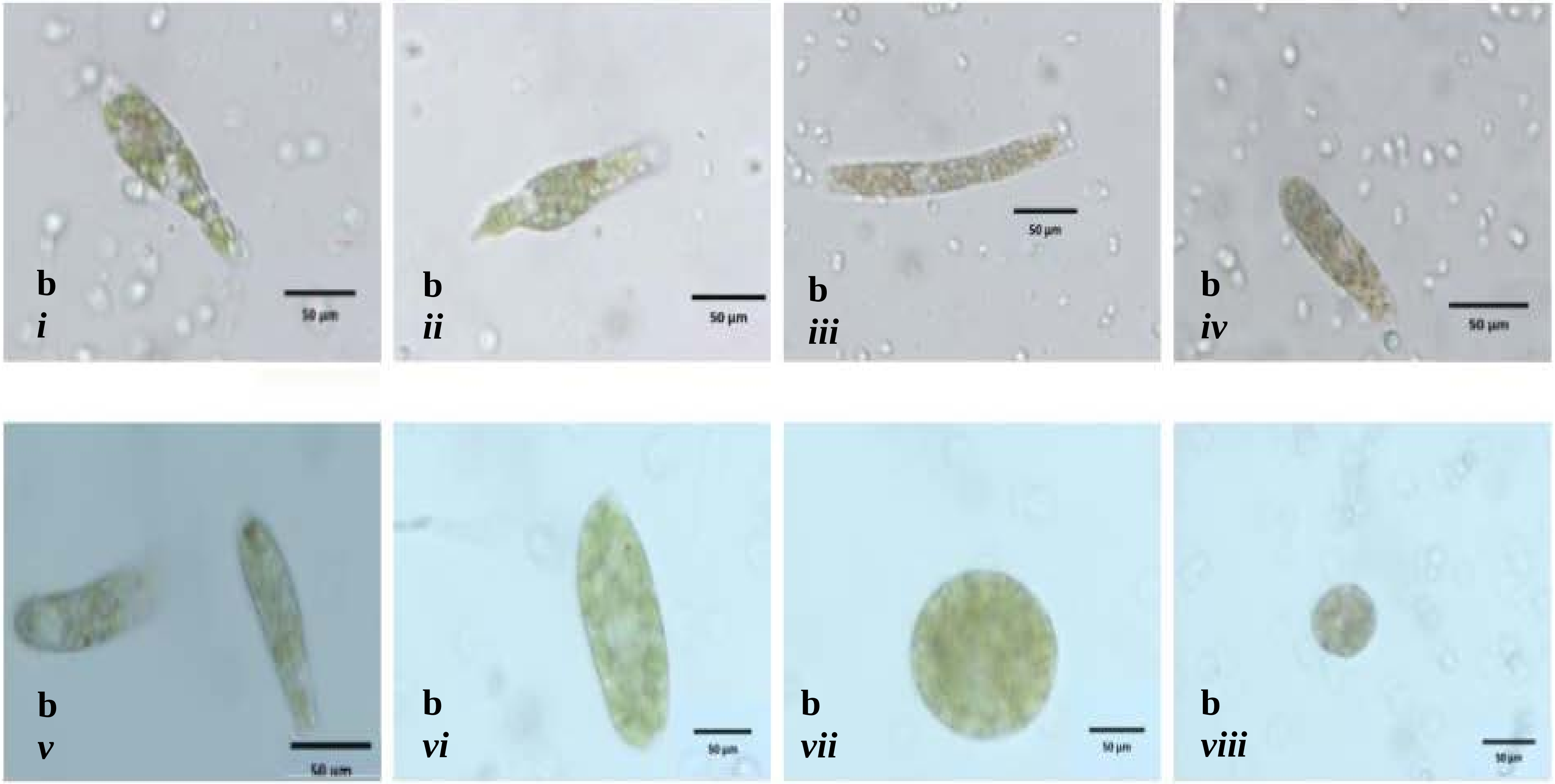

b) *E. gracilis* grown in MAM for 8 days with i) 6 mM SO_4, ii) 30 mM SO_4, iii) 60 mM SO_4, iv) 120 mM SO_4 or grown in MAM for 10 days with v) 8 mM NO_3 vi) 38 mM NO_3 vii) 76 mM NO_3, and viii) 151 mM NO_3. Photos were taken at 40x magnification. The scale bar is 50µm in all images.

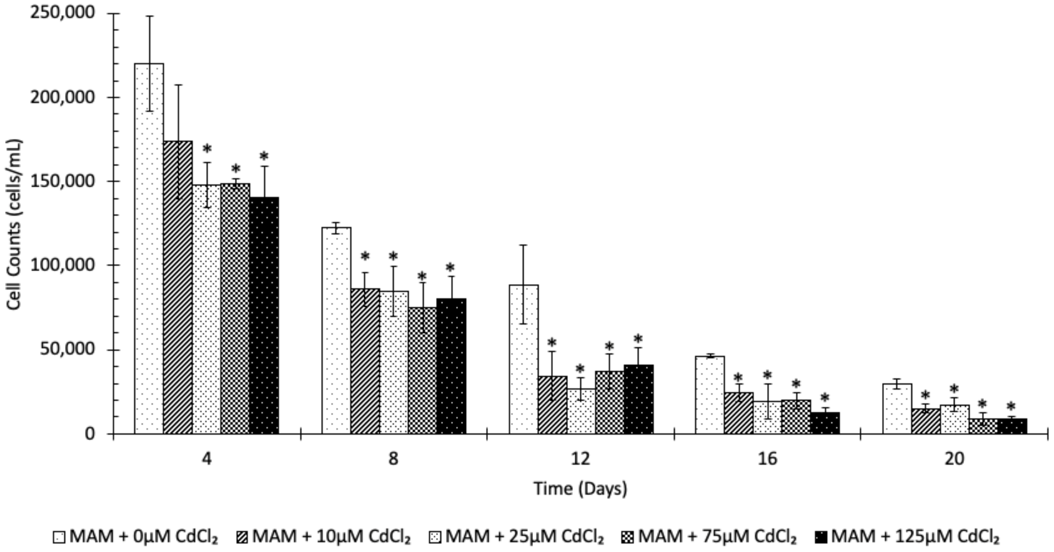
250,000
200,000
150,000
100,000
50,000
0
Cell Counts (cells/mL)
4
8
12
16
20
Time (Days)
MAM + 0μM CdCl2
MAM + 10μM CdCl2
MAM + 25μM CdCl2
MAM + 75μM CdCl2
MAM + 125μM CdCl2

a

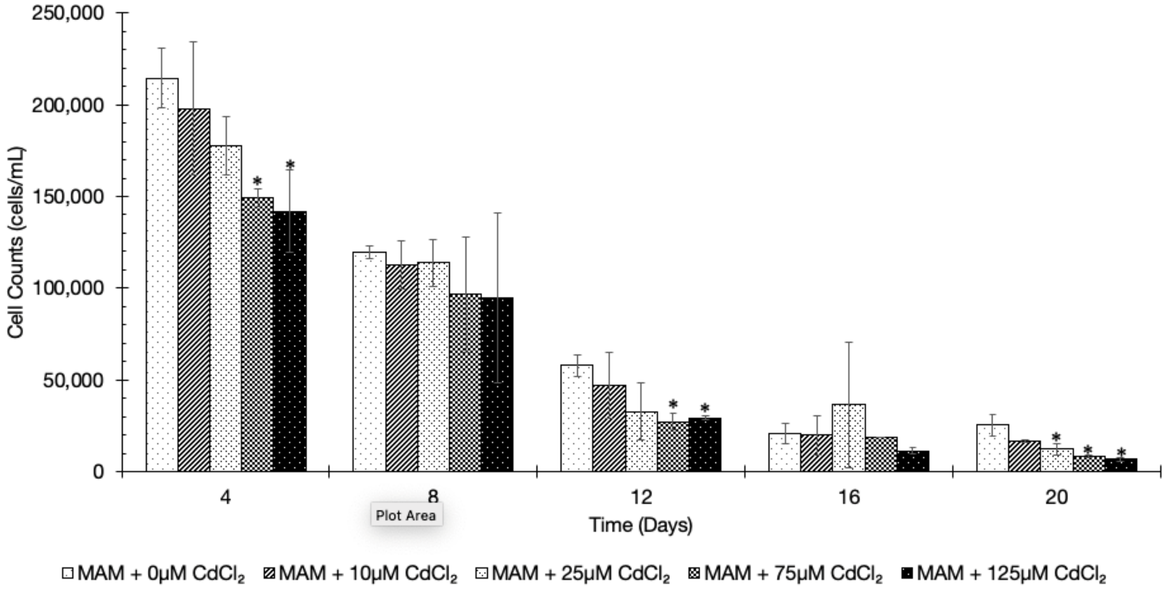
250,000
200,000
150,000
100,000
50,000
0
Cell Counts (cells/mL)
4
8
12
16
20
Plot Area
Time (Days)
MAM + 0μM CdCl2
MAM + 10μM CdCl2
MAM + 25μM CdCl2
MAM + 75μM CdCl2
MAM + 125μM CdCl2

b

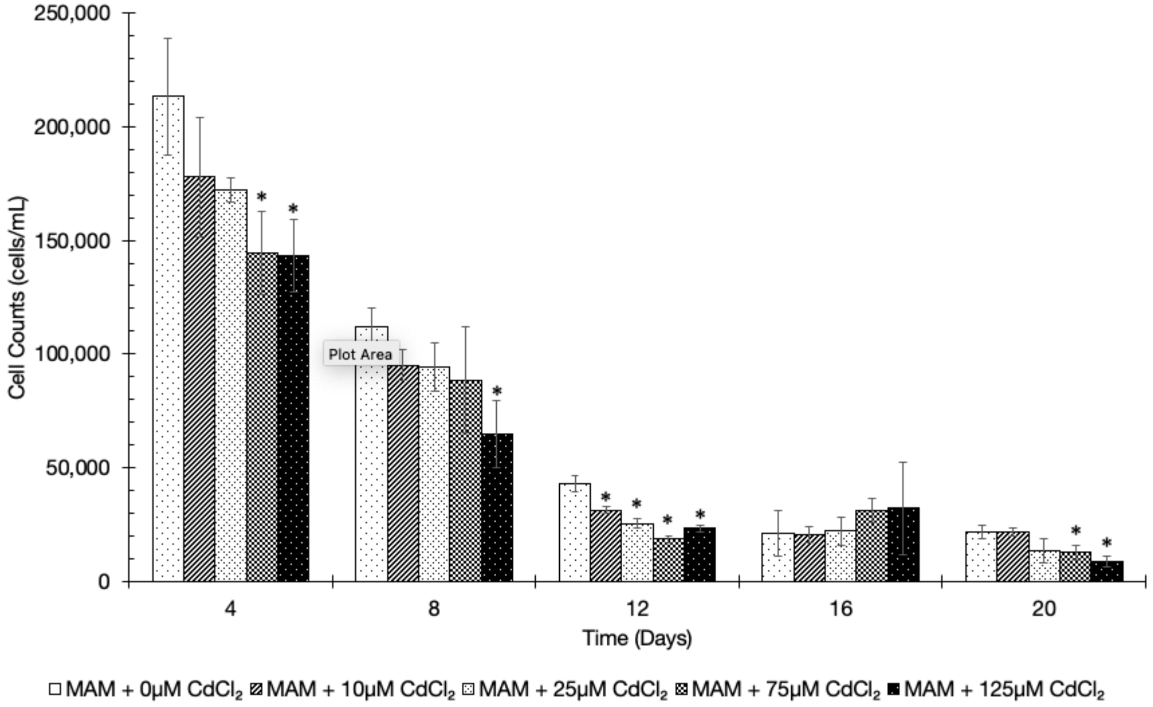

c

Figure S2: Assessment of various concentrations of Cd on *E. gracilis*. Time course exposure of *E. gracilis* exposed to various concentrations of $CdCl_2$ following 44 days of growth in MAM (a), pretreatment in elevated S (b), and pretreatment in elevated N (c). Bars on each graph represent the number of viable cells in each culture (cells/mL). Statistical difference between control (0 µM $CdCl_2$) and Cd exposed cells in each treatment group were assessed using a t-test and significant difference between cells count are indicated by an asterisks (* = $p < 0.05$). Error bars represent the standard deviation between viable cell counts of the biological replicates ($n = 3$). Differences between treatment groups indicate that while control cells display sensitivity to as low as 25 µM $CdCl_2$ after 4 days of exposure, cells treated with elevated S or N do no exhibit sensitivity to 25 µM $CdCl_2$ until 20 and 12 days, respectively. To assess potential molecular differences between cells that were pretreated before Cd exposure and those that were not, the concentration of 25 µM $CdCl_2$ was selected for subsequent experiments. The Cd exposure period of 8 days was also selected for subsequent experiments to ensure enough viable cells were available for RNA isolations and RNA-sequencing.

Table S1: Average total number of raw and trimmed reads across 6 biological replicates of each RNA-Seq library.

Library	Number of Raw reads	Number of Trimmed Reads
Control	22,474,149	18,867,533
Control + 25 µM $CdCl_2$	22,938,670	19,041,189
$MgSO_4$	21,179,800	17,495,371
$MgSO_4$ + 25 µM $CdCl_2$	21,329,961	17,848,454
NH_4NO_3	26,431,615	22,355,220
NH_4NO_3 + 25 µM $CdCl_2$	25,430,061	21,699,217

Table S2: Average total number of reads mapped to de novo assembly using bowtie2 (v2.4.2).

Library	Number of Reads Mapped
Control	33,479,732
Control + 25 µM $CdCl_2$	33,516,640
$MgSO_4$	28,305,301
$MgSO_4$ + 25 µM $CdCl_2$	31,105,360
NH_4NO_3	39,336,238
NH_4NO_3 + 25 µM $CdCl_2$	38,067,499

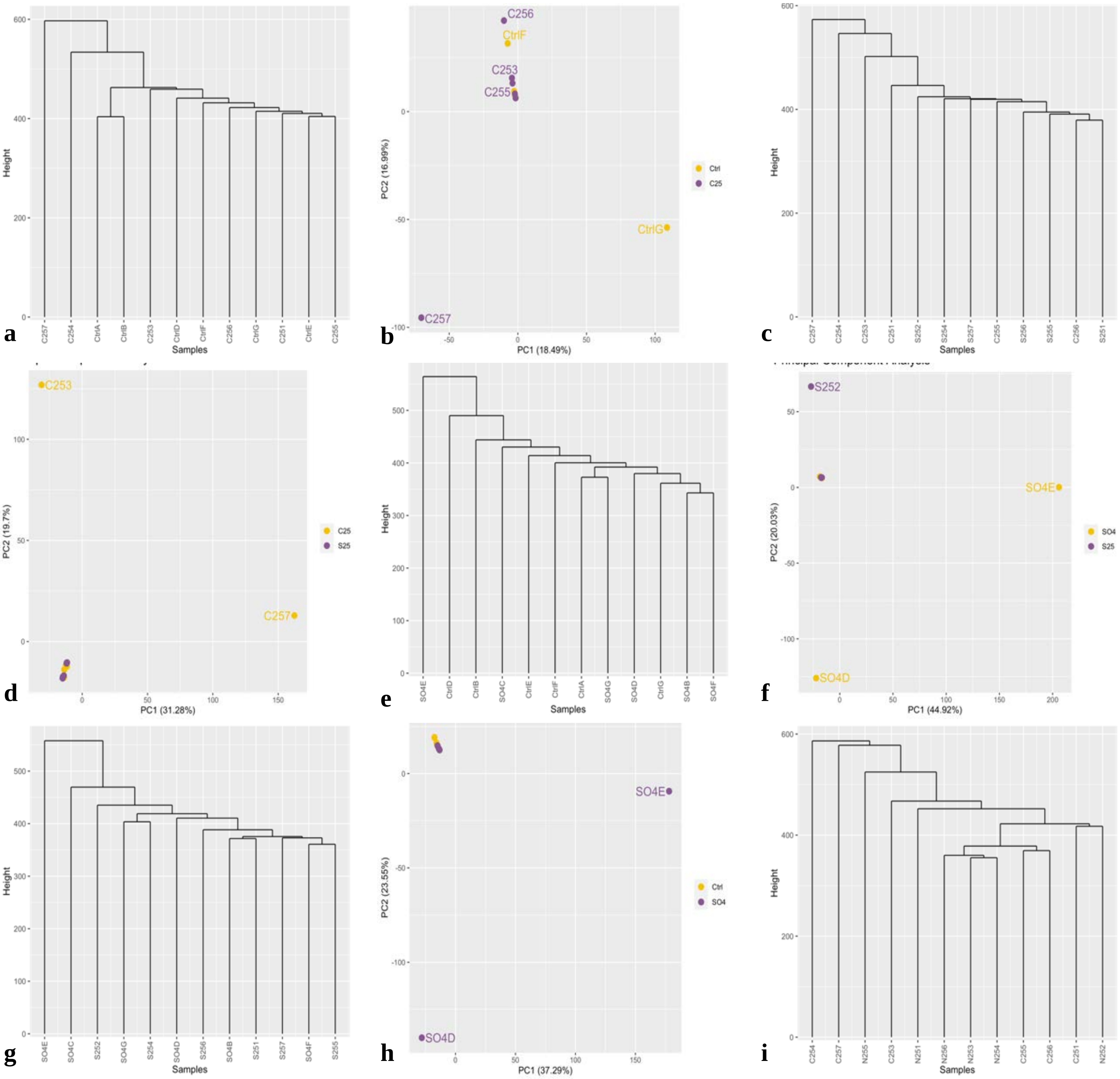
a
Height
Samples
C257
C254
CtrlA
CtrlB
C253
CtrlD
CtrlF
C256
CtrlG
C251
CtrlE
C255
b
PC1 (18.49%)
PC2 (16.99%)
C256
CtrlF
C253
C255
CtrlG
C257
Ctrl
C25
c
Height
Samples
C257
C254
C253
C251
S252
S254
S257
C255
S256
S255
C256
S251
d
PC1 (31.28%)
PC2 (19.7%)
C253
C257
C25
S25
e
Height
Samples
SO4E
CtrlD
CtrlB
SO4C
CtrlE
CtrlF
CtrlA
SO4G
SO4D
CtrlG
SO4B
SO4F
f
PC1 (44.92%)
PC2 (20.03%)
S252
SO4E
SO4D
SO4
S25
g
Height
Samples
SO4E
SO4C
S252
SO4G
S254
SO4D
S256
SO4B
S251
S257
SO4F
S255
h
PC1 (37.29%)
PC2 (23.55%)
SO4E
SO4D
Ctrl
SO4
i
Height
Samples
C254
C257
N255
C253
N251
N256
N253
N254
C255
C256
C251
N252

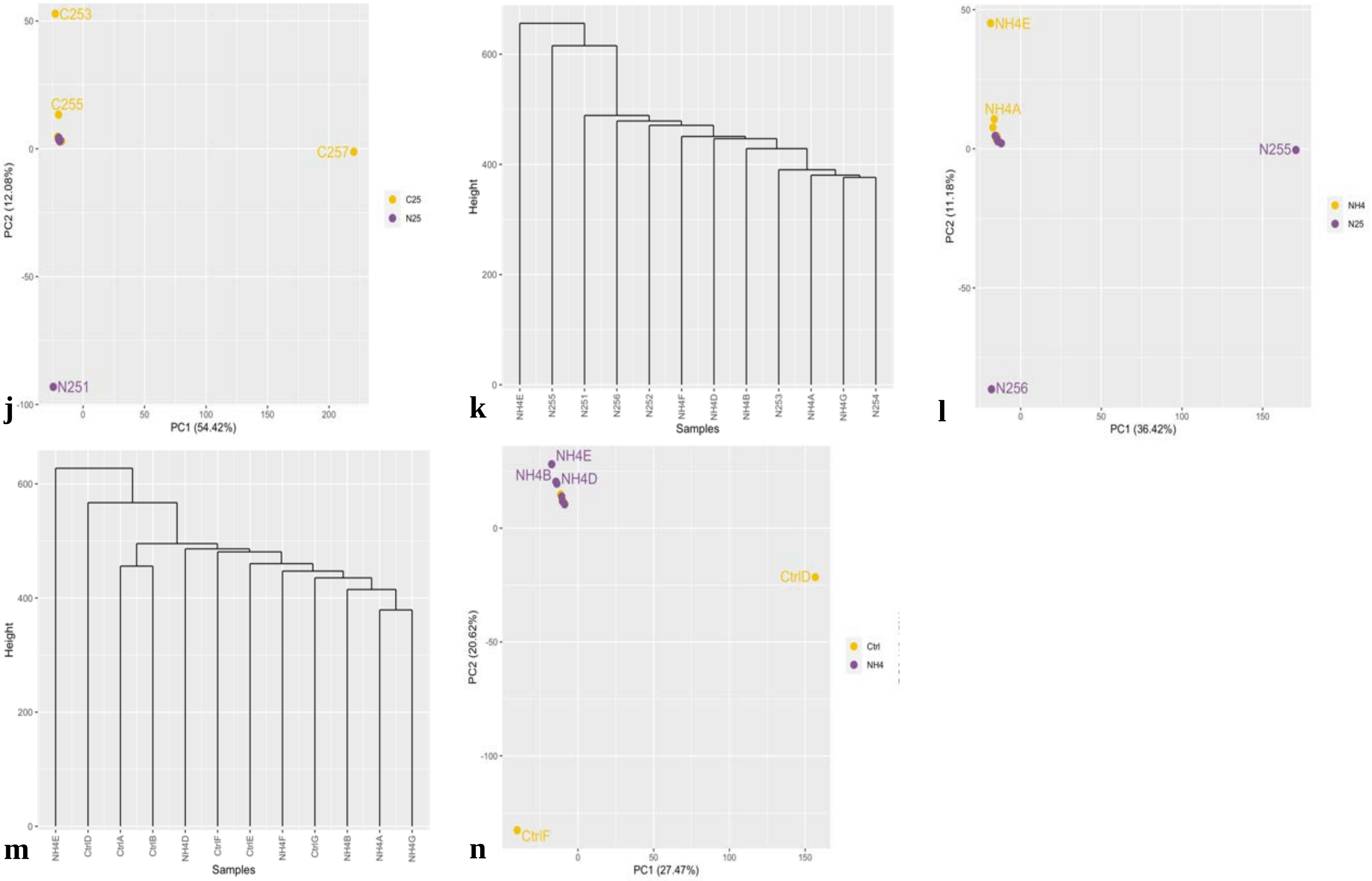

Figure S3: Clustering analysis showed no separation between control (MAM), S-pretreated, and N-pretreated *E. gracilis* cultures with or without $CdCl_2$. a-b) Clustering analysis done using a dendrogram (a) and principal component analysis (b), (MAM = yellow circles; MAM + 25 µM $CdCl_2$ = purple circles). c-d) dendrogram (c) and principal component analysis (d), (MAM + 25 µM $CdCl_2$= yellow circles; S + 25 µM $CdCl_2$ = purple circles). e-f) dendrogram (e) and principal component analysis (f), (S = yellow circles; S + 25 µM $CdCl_2$ = purple circles). g-h) dendrogam (g) and principal component analysis (h), (MAM = yellow circles; S = purple circles). i-j) dendrogram (i) and principal component analysis (j), (MAM + 25 µM $CdCl_2$ = yellow circles; N + 25 µM $CdCl_2$ = purple circles). k-l) dendrogram (k) and principal component analysis (l), (N= yellow circles; N+ 25 µM $CdCl_2$ = purple circles). m-n) dendrogram (m) and principal component analysis (n), (MAM = yellow circles; N = purple circles).

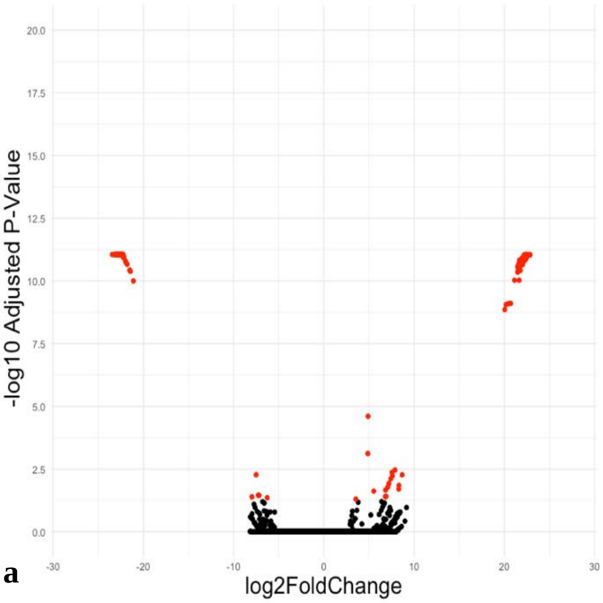
-log10 Adjusted P-Value
log2FoldChange

a

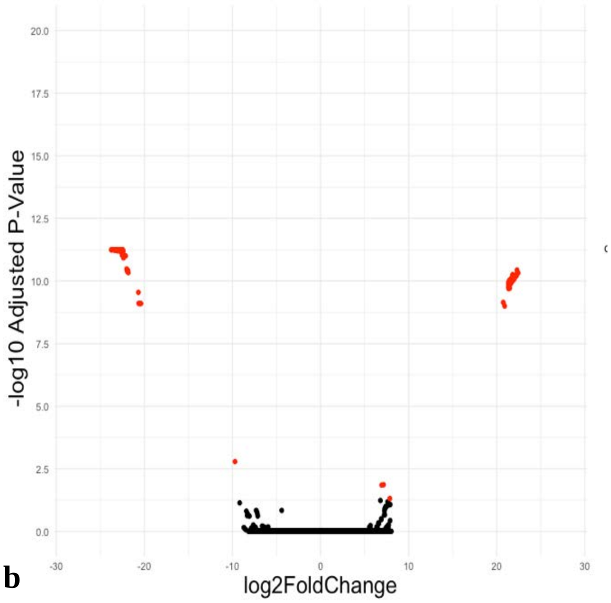
-log10 Adjusted P-Value
log2FoldChange

b

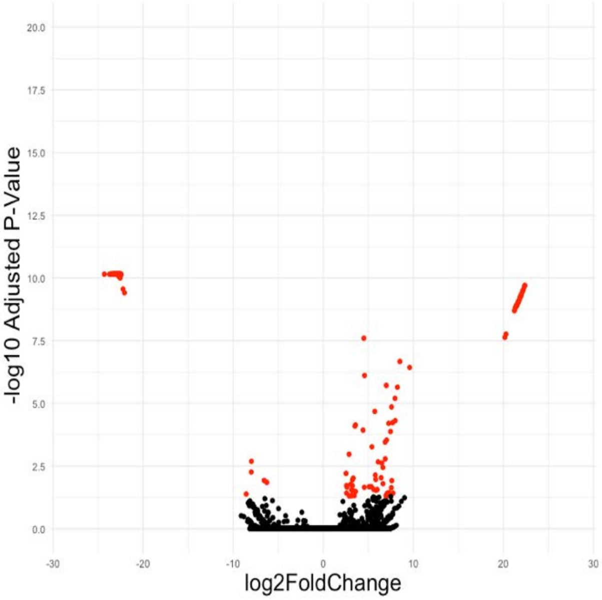
-log10 Adjusted P-Value
log2FoldChange

c

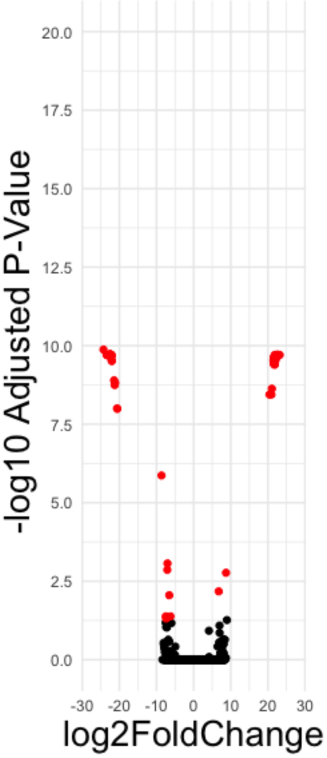
-log10 Adjusted P-Value
log2FoldChange

d

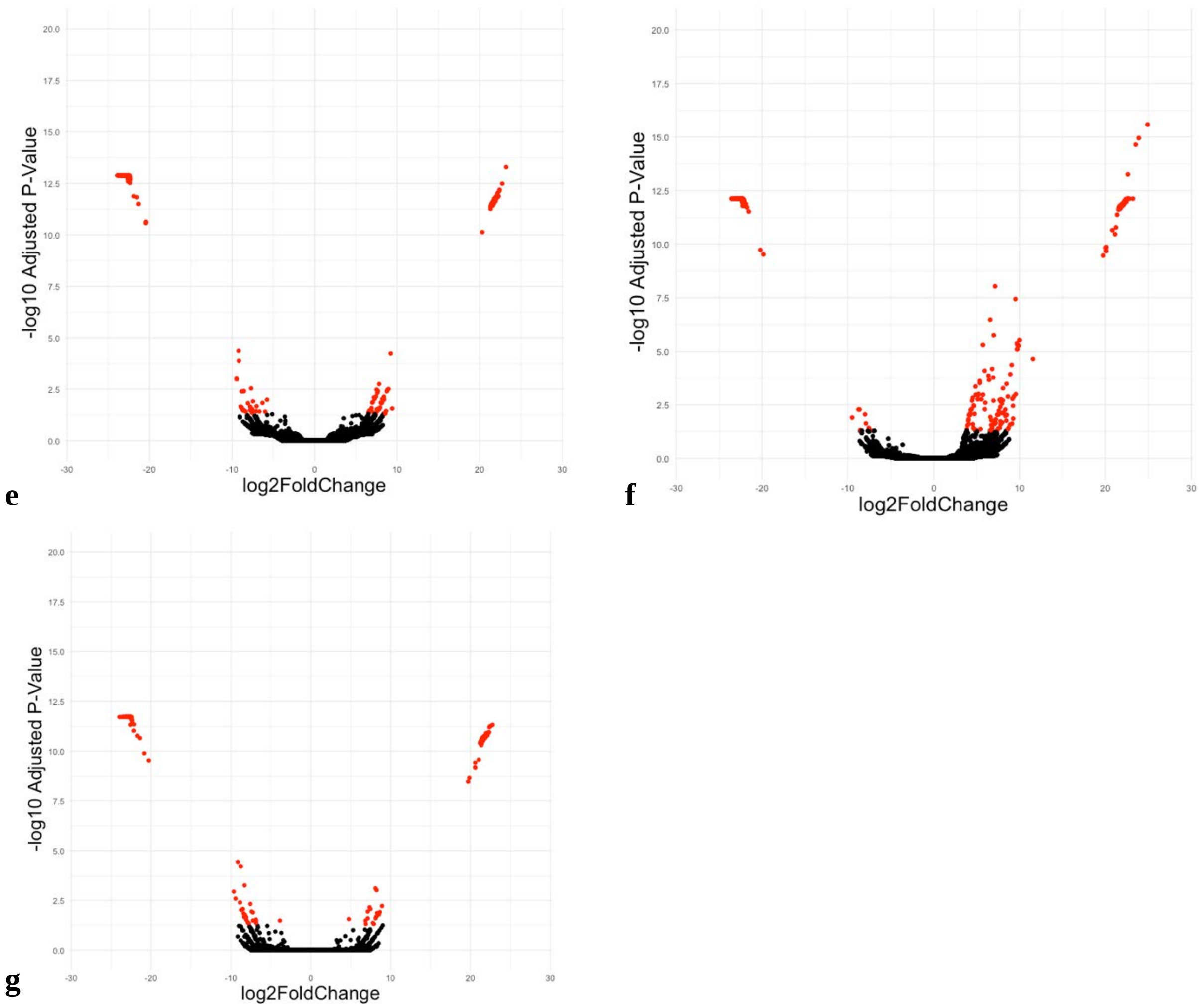

Figure S4: DGE analysis of control (MAM), S-pretreated, and N-pretreated *E. gracilis* cultures grown in the presence and absence of Cd shows a difference in transcript levels. Differential transcript expression was assessed using DESEq2 with a significance level of 0.05 (>2 or <-2) across control, S-pretreated, and N-pretreated *E. gracilis* cultures with and without $CdCl_2$ exposure. a-g) Volcano plots comparing the -log10 adjusted p-value (y-axis) to the log2 fold change (x- axis) between a) Control cultures with and without $CdCl_2$, b) S-pretreated cultures vs control cultures with $CdCl_2$, c) S-pretreated cultures with and without $CdCl_2$, d) S-pretreated cultures vs control cultures, e) N-pretreated cultures vs control cultures both with $CdCl_2$, f) N-pretreated cultures in the presence and absence of $CdCl_2$, and g) N-pretreated cultures vs control cultures. Red dots indicate genes that are differentially expressed and statistically significant, while black dots indicate genes whose expression was not significantly different.

Table S3: GO term analysis results for control, S-pretreated, and N-pretreated *E. gracilis* cultures.

	GO-Term	Annotation Set	Fold-Enrichment	P-Value	Database
Non-pretreated without $CdCl_2$ vs non-pretreated with $CdCl_2$	Positive regulation of proteasomal protein catabolic process (GO:1901800)	Biological Process	>100	4.38 E-5	*Chlamydomonas reinhardtii*
	Phosphotransferase activity carboxyl group as acceptor (GO:0016774)	Molecular Function	>100	8.90 E-6	*Arabidopsis thaliana*
S-pretreated exposed to $CdCl_2$ vs non-pretreated exposed to $CdCl_2$	Cysteine metabolic process (GO:0006534)	Biological Process	>100	9.79 E-6	*Trypanosoma brucei*
S-pretreated without $CdCl_2$ vs S-pretreated with $CdCl_2$	Catalytic activity (GO:0003824)	Molecular Function	3.13	1.42 E-6	*Arabidopsis thaliana*
N-pretreated exposed to $CdCl_2$ vs non-pretreated exposed to $CdCl_2$	Translational elongation (GO:0006414)	Biological Process	>100	4.00 E-8	*Arabidopsis thaliana*

Table S4: DGE results for gene encoding proteins related to respiration and transmembrane transport in S-treated and N-treated *E. gracilis* cultures in the presence and absence of $CdCl_2$

Treatment	Gene Encoded protein	Transcript level change	Relationship to Cd exposure
S-treated cultures in the presence and absence of Cd	NADH dehydrogenase<br><br>NADH-ubiquinone oxidoreductase	Decreased<br><br>Decreased	It is possible that growth in elevated S not only promoted the growth of *E. gracilis* (Figure 1), but also mitigated the effects of Cd. A similar correlation was observed in the microalgae *Chlamydomonas moewusii* which demonstrated higher Cd tolerances when grown in media containing higher concentrations of sulphate [66].
N-treated cultures in the presence and absence of Cd	NADH dehydrogenase<br><br>NADH-ubiquinone oxidoreductase	Increased<br><br>Increased	This response to N level but not Cd differs from the oxidative stress responses observed in plants as a result of Cd toxicity [3,67]
	PsaD in photosystem II	Decreased	This response to N level but not Cd differs from the oxidative stress responses observed in plants as a result of Cd toxicity [3,67]. However, the decreased transcript levels observed for *E. gracilis* PsaD in photosystem II of N pretreated cultures exposed to $CdCl_2$ relative to control culture exposed to $CdCl_2$ suggest some impact on photosynthesis.<br><br>Future investigations may better elucidate the impact on photosynthesis observed here and since photosynthesis and respiration are coupled in *E. gracilis*, these findings may indicate that subjecting *E. gracilis* to elevated N and Cd also impacts respiration [68].

S-treated and N-treated cultures exposed to Cd	ABC transporter		This suggests that *E. gracilis* may be altering intracellular Cd levels by varying its sequestration, intracellular distribution, or excretion. ABC transporters confer heavy metal tolerance in the model fungi *Saccharomyces cerevisae* and *Schizosaccharomyces pombe* [69]. It has also hypothesised that these ABC transport proteins may be responsible for the regulation of Cd levels in the placenta in mammalian cells [70]. Since there was an altered response in *E. gracilis* with pre-treatment in elevated levels of either S or N, our data suggests that the alteration of Cd flow through its cells is modulated by nutritional state or other processes associated with S and N metabolism. This modulation is predicted to be implemented through an impact on ABC transporter gene expression.
